# Current perspectives and trends in nanoparticle drug delivery systems in breast cancer: bibliometric analysis and review

**DOI:** 10.3389/fbioe.2023.1253048

**Published:** 2023-09-12

**Authors:** Sheng Sun, Ye-hui Wang, Xiang Gao, He-yong Wang, Lu Zhang, Na Wang, Chun-mei Li, Shao-quan Xiong

**Affiliations:** ^1^ Sichuan Integrative Medicine Hospital, Chengdu, China; ^2^ Chengdu University of Traditional Chinese Medicine, Chengdu, China

**Keywords:** bibliometrics, visualization, breast cancer, nanoparticle drug delivery systems, nanoparticles

## Abstract

The treatment of breast cancer (BC) is a serious challenge due to its heterogeneous nature, multidrug resistance (MDR), and limited therapeutic options. Nanoparticle-based drug delivery systems (NDDSs) represent a promising tool for overcoming toxicity and chemotherapy drug resistance in BC treatment. No bibliometric studies have yet been published on the research landscape of NDDS-based treatment of BC. In this review, we extracted data from 1,752 articles on NDDS-based treatment of BC published between 2012 and 2022 from the Web of Science Core Collection (WOSCC) database. VOSviewer, CiteSpace, and some online platforms were used for bibliometric analysis and visualization. Publication trends were initially observed: in terms of geographical distribution, China and the United States had the most papers on this subject. The highest contributing institution was Sichuan University. In terms of authorship and co-cited authorship, the most prolific author was Yu Zhang. Furthermore, Qiang Zhang and co-workers have made tremendous achievements in the field of NDDS-based BC treatment. The article titled “Nanomedicine in cancer therapy: challenges, opportunities, and clinical applications” had the most citations. The *Journal of Controlled Release* was one of the most active publishers in the field. “Global cancer statistics 2018: GLOBOCAN estimates of incidence and mortality worldwide for 36 cancers in 185 countries” was the most cited reference. We also analysed “hot” and cutting-edge research for NDDSs in BC treatment. There were nine topic clusters: “tumour microenvironment,” “nanoparticles (drug delivery),” “breast cancer/triple-negative breast cancer,” “combination therapy,” “drug release (pathway),” “multidrug resistance,” “recent advance,” “targeted drug delivery”, and “cancer nanomedicine.” We also reviewed the core themes of research. In summary, this article reviewed the application of NDDSs in the treatment of BC.

## 1 Introduction

**FIGURE 1:**
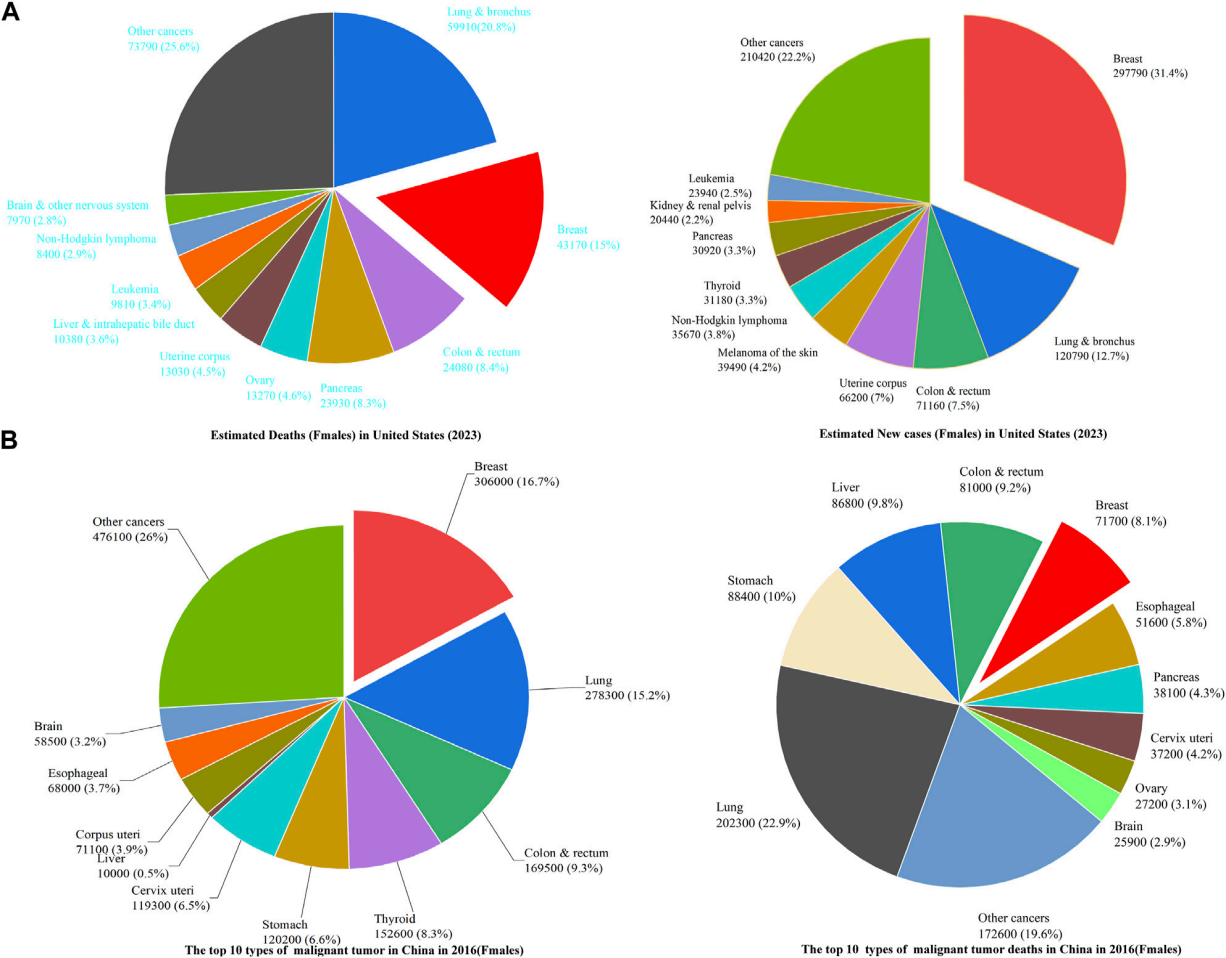
Epidemiological overview of breast cancer in the United States and in China.

Breast cancer (BC) is a global, life-threatening cancer. An estimated 2.1 million female patients worldwide were reported to have BC in 2018, accounting for 25% of cancer cases among women (Bray et al., 2018). As shown in Figure 1A, BC is projected to account for 31% of new cancers in female patients and 15% of deaths for new cancers in women in the United States in 2023 (Siegel et al., 2023). According to recent statistics, the incidence of female cancer cases is increasing in China. In 2016, approximately 1,829,600 female cancer cases and 882,800 cancer-related deaths among women were reported in rural and urban regions of China (Zheng et al., 2023). BC was the most common cancer and the fifth leading cause of cancer-related death among women in China in 2016 (Zheng et al., 2023) (Figure 1B).

Despite substantial improvements in early diagnosis and effective treatment strategies, recurrence and metastasis (i.e., bone 75%; liver, pleura or lung, and brain 15%–30%) remain considerable threats to the survival of BC patients (Ruiterkamp and Ernst, 2011; Liang et al., 2020). Triple-negative BC (TNBC) is also associated with a poor prognosis (Li et al., 2019). Nowadays, therapeutic options for BC are limited and include surgery, chemotherapy, immunotherapy, endocrine therapy, targeted therapy, and radiation therapy (Aniogo et al., 2019; Pondé et al., 2019). Chemotherapeutic drugs generally show high toxicity, low bioavailability, weak water solubility, and moderate biological diffusion, and such drugs lack tumour-targeting activity (Peng et al., 2017; Pondé et al., 2019; Pei et al., 2022; Vyas et al., 2022).

It is necessary to develop more ideal targeted drug delivery systems for the effective treatment of BC (Lammers et al., 2012). The field of nanomedicine is almost exclusively focused on tumour-targeted drug delivery (Wang et al., 2021a). Nanoparticle drug delivery systems (NDDSs) allow the introduction of therapy based on nanotechnology in the body by regulating the location, rate, and time of delivery (Khan et al., 2022). NDDSs have been extensively investigated owing to the advantages of reduced toxicity, prolonged drug action, high drug bioavailability, improved pharmacokinetic properties, and efficacy (Kang et al., 2018; Patra et al., 2018; Kaushik et al., 2022). The progress of nanotechnology and chemical/pharmaceutical engineering has led to the development of various drug delivery systems (DDSs).

VOSviewer and CiteSpace were used for data visualization (van Eck and Waltman, 2010), and bibliometric analysis has been used in the field of nanomaterials (Zhu et al., 2021). However, there have been few bibliometric studies on the application of NDDSs in the treatment of BC. In this review, we used numerous platforms to conduct bibliometric analysis on articles from 1 January 2012 to 31 December 2022. We examined the annual trends of publication, countries/regions, institutions, authors and co-cited authors, distribution of highly cited literature, co-cited references, and keywords. The study aims to solve the following problems:

Q1: What global developmental trends in research have occurred in the field of NDDS-based BC treatment?

Q2: Which countries/regions, institutions, and authors have been most productive in the field to date, based on scientific collaboration networks?

Q3: What are the top 10 most highly cited papers, the most preferred journals, and the top-yielding co-cited references in the field? What have been the main research directions in the field based on co-citation analysis, and how have they changed over time?

Q4: What are the main research directions and hotspots of keyword analysis?

Q5: What cutting-edge research will develop in the near future?

This article aims to help both newcomers and specialists identify the breadth of the field of NDDS-based BC treatment and propose novel, important topics of interest in a visual manner. We also present a review of the literature concerning the major aspects and the evolution of NDDSs in BC treatment.

## 2 Materials and methods

### 2.1 Data extraction and collection

Scopus and Web of Science (WOS) were the main primary databases used for bibliometric studies (Sood et al., 2022). We chose the WOS Core Collection (WOSCC) database. Only articles and reviews were analysed. Two researchers conducted the search independently according to the search formula (MESH terms): #1: TI=(“Nanoparticle Drug Delivery System” OR “Nanoparticle Based Drug Delivery System*” OR “Nano Delivery System*”); #2: TI=(“Breast Neoplasms” OR “Breast Tumour*” OR “Tumour, Breast*” OR “Human Mammary Carcinoma*” OR “Breast Carcinoma*”) published and indexed between 1 January 2012 and 31 December 2022. Because of update delays in WOS, items not published or indexed from 2012–2022 are not discussed in the paper. The final datasets were “#1 AND #2” and the wildcard character (*) was used to capture data sources. The search date was 13 May 2023, and the language was limited to English. Supplementary Figure S1A shows the procedure for data retrieval and collection.

### 2.2 Statistics and analysis

#### 2.2.1 Bibliometric analysis

Numerous platforms were used to analyse and visualize WOSCC, such as the online analysis platform of bibliometrics (https://bibliometric.com/app, https://webofscience.com/wos), CiteSpace, and VOSviewer (Chen, 2004; Sood and Rawat, 2021). These were used to visualize and extract information from the collected database.

CiteSpace, a Java-based bibliometric application, was used to analyse dynamics and research clusters in preferred science topics (Chen, 2004) as it is compatible with the WOS data format. CiteSpace (version 6.2. R2) was used to visualize the distribution of countries/regions, contributions of institutions, authors, co-cited references, and co-occurring subject categories, and create a dual-map overlay of journals, the timeline view map, keyword analysis, and keyword and reference bursts.

VOSviewer is software that creates bibliometric maps (van Eck and Waltman, 2010). Three different visualization maps with different meanings were generated by VOSviewer, including network, density, and overlay visualization. VOSviewer (version 1.6.17) was used to visualize country/regional cooperation, author co-authorship, and keyword co-occurrence. A node on maps represents a specific parameter. Its size is decided by weighing the attributes. The clusters, links, and level of total link strength (TLS) reflect the strength of connections; TLS represents the level of strength between the connection nodes, which is weighted by the thickness of the lines.

We collected studies, annual publications, countries/regions, institutions, authors, articles, journals, references, and keywords. Retrieved articles and reviews were saved as plain text files and exported to EndNote Desktop as full-text citation records, ultimately called “download_XXX.Txt.”

### 2.3 Statistical analysis

Microsoft Excel 2016, R software (V4.3.0.), and SPSS (IBM SPSS Statistics 19.0) were used to plot graphs and descriptive statistics. Microsoft Excel was used for numerical analysis and drawing graphs. Pearson’s correlation coefficient was selected to test the correlation between publications and citations using SPSS. Supplementary Figure S1B presents the research framework.

## 3 Results

### 3.1 Annual publication analysis

**FIGURE 2:**
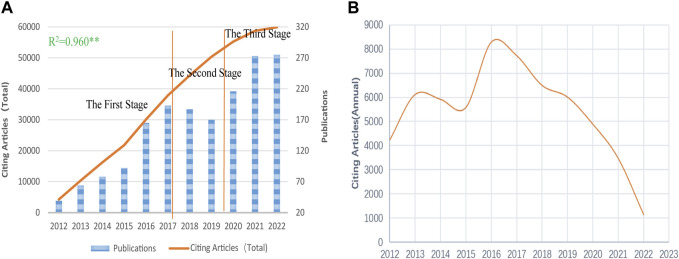
**(A)** Increasing trend in publications and total citing articles (without self-citations); statistics evaluated using Pearson’s test; ** significant correlation coefficient. **(B)** Citing article (annual) trends on NDDSs in BC research from 2012 to 2022.

A total of 1,752 papers published and indexed in NDDSs on BC research were obtained by WOS between 1 January 2012 and 31 December 2022, including 1,379 (78.71%) articles and 373 reviews (21.29%). Figure 2A shows the increasing trend in publications and total citing articles (without self-citations) for NDDSs in BC research, with a significant correlation coefficient (*R* ^2^ = 0.960). Research and development have advanced rapidly, with more than half of all publications in the last 5 years. The number of global articles per annum has risen from 39 in 2012 to 275 in 2022, with a 55.01% annual growth rate. Before 2016, less than 100 publications were published annually. After that, it quickly grew to 275 by 2022, suggesting rapid growth. All papers were cited in 59,869 articles (without self-citations as of the search date), with 34.17 average citations per item (Figure 2A). Figure 2B exhibits a downtrend after 2016, which needs further analysis.

### 3.2 Country/region analysis

**FIGURE 3:**
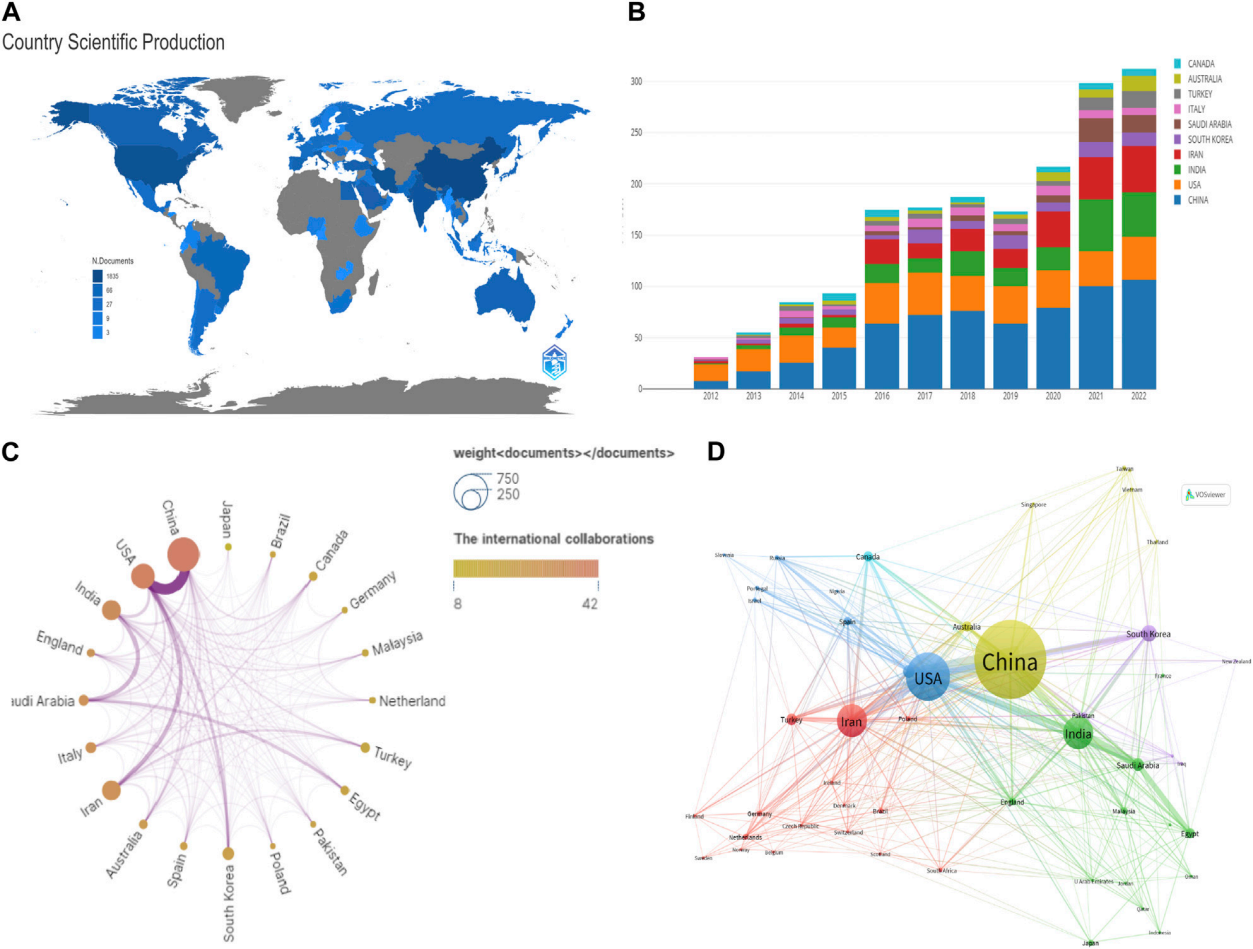
**(A)** Distribution of research on NDDSs in BC based on total worldwide publications. **(B)** Changing trend in the top 10 countries/regions based on annual publication 2012–2022. **(C)** Network visualization map of countries/regions. **(D)** Network visualization map of citation countries/regions produced by VOSviewer; line thickness denotes citation strength.

According to bibliometric analysis, the research on NDDSs in BC was conducted in 336 different countries/regions (Figure 3A). Supplementary Table S1 lists the top ten countries/regions by the number of publications produced. China ranks first, with the United States, Iran, and India also having more than 500 publications; Canada had more than 116. Among the top 10 countries, papers were primarily most published in Asia (Supplementary Table S1). The United States had the strongest TLS, followed by China.

Figure 3A shows that papers were primarily distributed around the world. Annual publications in countries/regions over 2012–2022 are shown in Figure 3B. China had the highest annual publication (36.69%, 1835), followed by the United States (17.39%, 870) and Iran (17.13%, 857). Figure 3C indicates international cooperation between countries/regions. The United States enjoyed cooperation from many countries, working in close cooperation with China, Iran, and South Korea. The VOSviewer showed global cooperation, with 76 nations included in our analysis (Figure 3D). It was evident that the top five TLS were the United States (TLS = 269), China (TLS = 204), India (TLS = 125), Iran (TLS = 136), and Saudi Arabia (TLS = 78).

“MCP” is the number of papers with co-authors from other countries; “SCP” denotes the number of papers with co-authors from the same country (Pei et al., 2022). China had the highest SCP and MCP (Supplementary Table S1). Regarding international cooperation between countries and regions, China enjoyed the advantages of cooperation from other countries as well as domestic cooperation.

### 3.3 Contributions of institutions

**FIGURE 4:**
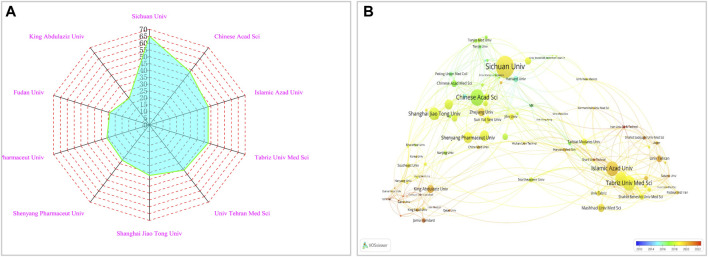
**(A)** Top ten institutions in the research field of NDDSs in BC. **(B)** Co-operation network between major institutions. In the visualization map (2012–2022), each node denotes one institution; lines between nodes represent co-citation relations. Different colours represent different times.

Contributions from 1982 institutions were recognized by the papers on NDDSs in BC research. Figure 4A includes the top 10 producing institutions according to publications. The H-index represents the importance and effect of the overall research contribution of a particular researcher. As shown in Supplementary Table S2, research institutions and universities were the main sources of research. The institution with the highest number of publications was Sichuan University (H-index, 30) with 65 articles and 2,819 total citations. Chinese Academy of Sciences (H-index, 37) was second. These two institutions contributed approximately 28.75% of the top 10 publications. Supplementary Table S2 shows Chinese Academy of Sciences had the highest average number of citations and the top H-index.

As seen in Figure 4B, VOSviewer software was used to show the collaboration network visualization map of the institutions. Tehran University of Medical Sciences (TLS = 71), Chinese Academy of Sciences (TLS = 72), and Islamic Azad University (TLS = 71) had the highest TLS. Chinese Academy of Sciences worked closely with numerous Chinese academic institutions. Islamic Azad University enjoyed cooperation from many countries but mainly worked in close cooperation with Tehran University of Medical Sciences.

### 3.4 Author and co-cited author analysis

To analyse influential researchers, we used the author’s contribution rate according to the Micro Scholar Social Networks–MSSN (Weigang et al., 2015). WOSCC metrics are also an important reference index (h-index, total citations, and publications) (Liu et al., 2021a). The survey included 9,414 authors by VOSviewer analysis. Supplementary Table S3 presents the ten most creative authors; Zhang, Yu (14 publications), Zhang, Qiang (11 publications), and Luo, Kui (11 publications) published the most papers. Among total citation rankings, Zhong-wei Gu was first (869). The highest author contribution rate for papers was Zhang, Yu (157.67), and the top H-index value was Qiang Zhang, Qiang (200).

**FIGURE 5:**
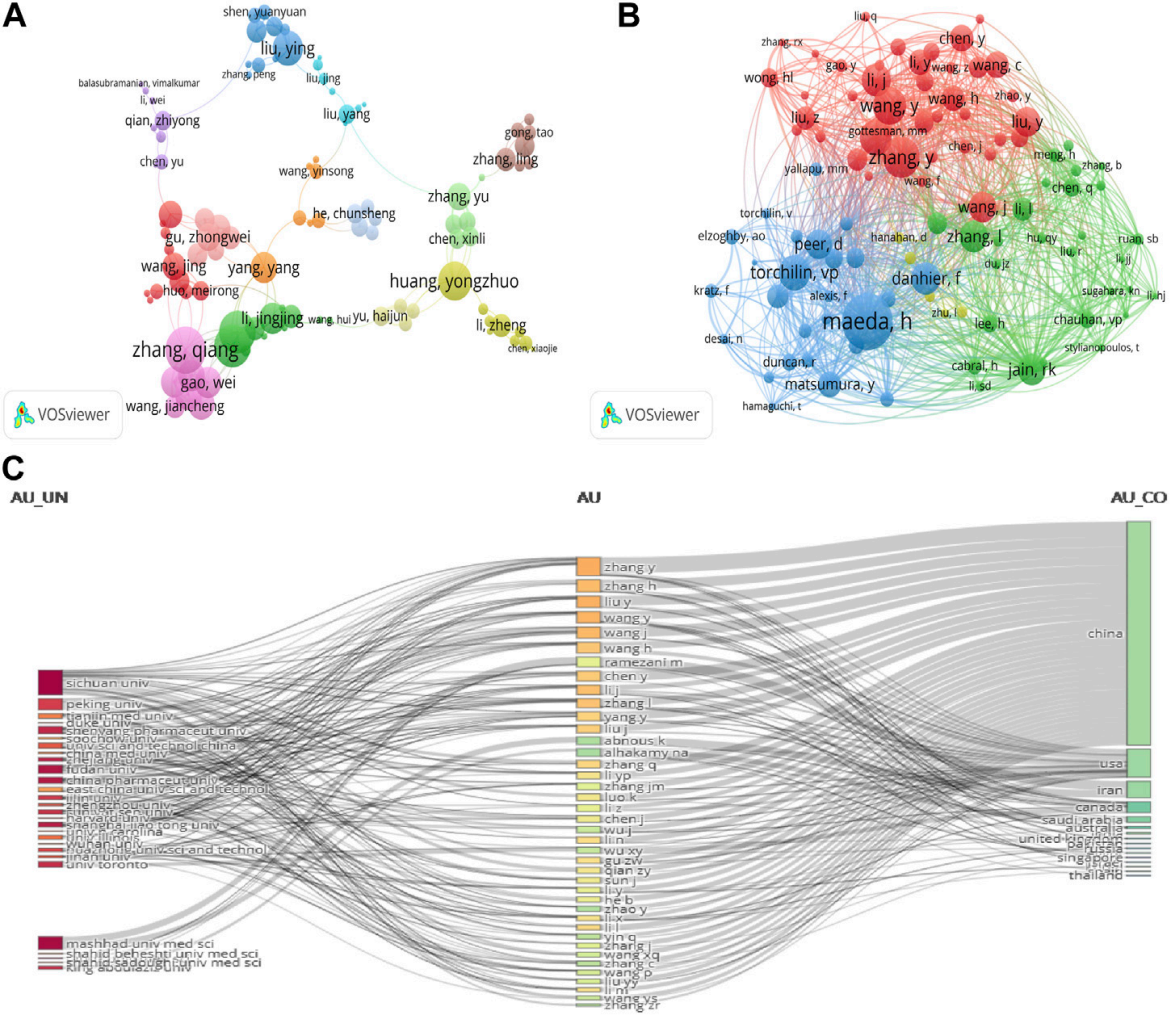
**(A)** Author co-authorship analysis generated using VOSviewer. In the network visualization, one cluster with the same colour indicates authors with a close relationship. **(B)** Collaborative network visualization of author citation analysis using VOSviewer; size of node shows the frequency of occurrence. Visualization map: each node represents an author. **(C)** Three-field plot represents the incoming and outgoing flows among top authors, affiliations, and countries contributing to research in the past 10 years.

In addition, VOSviewer was used to generate a cluster density map for co-authorship analysis. The threshold of the minimum number of documents for an author was set to 4, with 188 authors meeting the threshold. Authors were assigned to a cluster using the same, which denoted close cooperative relationships in this network visualization (Figure 5A); 13 author clusters were formed, weighted by TLS. Author clusters from China occupied the most. Figure 5A shows that Zhang, Qiang (45), Dai, Wen-bing, and Wang, Xue-qing (40) were team networks; the research focus of Zhang, Qiang’s team was highly homogeneous.

Figure 5B presents co-citation cited author analysis by VOSviewer. Thick lines indicate closer cooperation, while different colours indicate different clusters. Of the included 655 authors, more than 20 papers met the threshold, and 100 authors were selected. There were four author clusters: Zhang, L, and Shi, Ji, et al. (green); Hanahan, D and Wilhelm, S, et al. (red); Wang, Y and Zhang, Y, et al. (Pink); Maeda, H, and Torchillin, VP, et al. (yellow). Maeda, H, Zhang, Y, and Wang, Y were the top three authors—295, 216, and 196, respectively.

The three-field plot (Sankey diagram) analyses the relationship between different bibliometric indicators and constructs a comprehensive network graph of indicators. Between the fields, it presents the incoming and outgoing flows. The height of the rectangular nodes is linked to the frequency of occurrence in the network (Islam et al., 2022). To observe the outgoing and incoming flows among the authors, countries, and affiliations contributing to research over the last 10 years, a three-field plot was constructed. We selected “Affiliations” on the left, “Authors” in the middle field, and “Countries” on the right to draw the network relationship. The number of items was restricted to 40. Chinese authors mostly came from different affiliations. Zhang, Y’s incoming flow count was 12, making the outgoing flow count mostly 6 for China (Figure 5C).

### 3.5 Top articles and distribution of source journals

Supplementary Table S4A includes the ten articles with the most citations. With 1,260 citations according to WOSCC is “Nanomedicine in cancer therapy: challenges, opportunities, and clinical applications”, which was written by Wicki, Andreas. Additionally, the *Journal of Controlled Release* published 50% of the top 10 quoted original papers and had a significant scientific influence on academic performance in the field. All papers had funding organisations.

Some 366 academic journals published 1,752 papers. The *Journal of Controlled Release* (n = 73), *International Journal of Nanomedicine* (n = 72), *International Journal of Pharmaceutics* (n = 51), and *Biomaterials* (n = 49) were the top four academic publications (Supplementary Table S4B). Half of the 10 journal-publishing companies were in the Netherlands. Moreover, the top 10 active journals published 482 papers (accounting for 27.51%). Among the ten most prolific journals, *Biomaterials* had the highest IF of 15.304 and the greatest H-index (248). All journals in the top 10 were JCR Q1 or Q2. These findings suggest that the *Journal of Controlled Release* and *Biomaterials* has significantly advanced the topic.

Supplementary Table S4C presents the top 10 cited journals with the highest annual co-citation rate. The table shows that 50% of the top 10 cited journals’ publishing companies were in the Netherlands, whereas 40% were in the United States. All 10 journals fell into JCR Q1.

**FIGURE 6:**
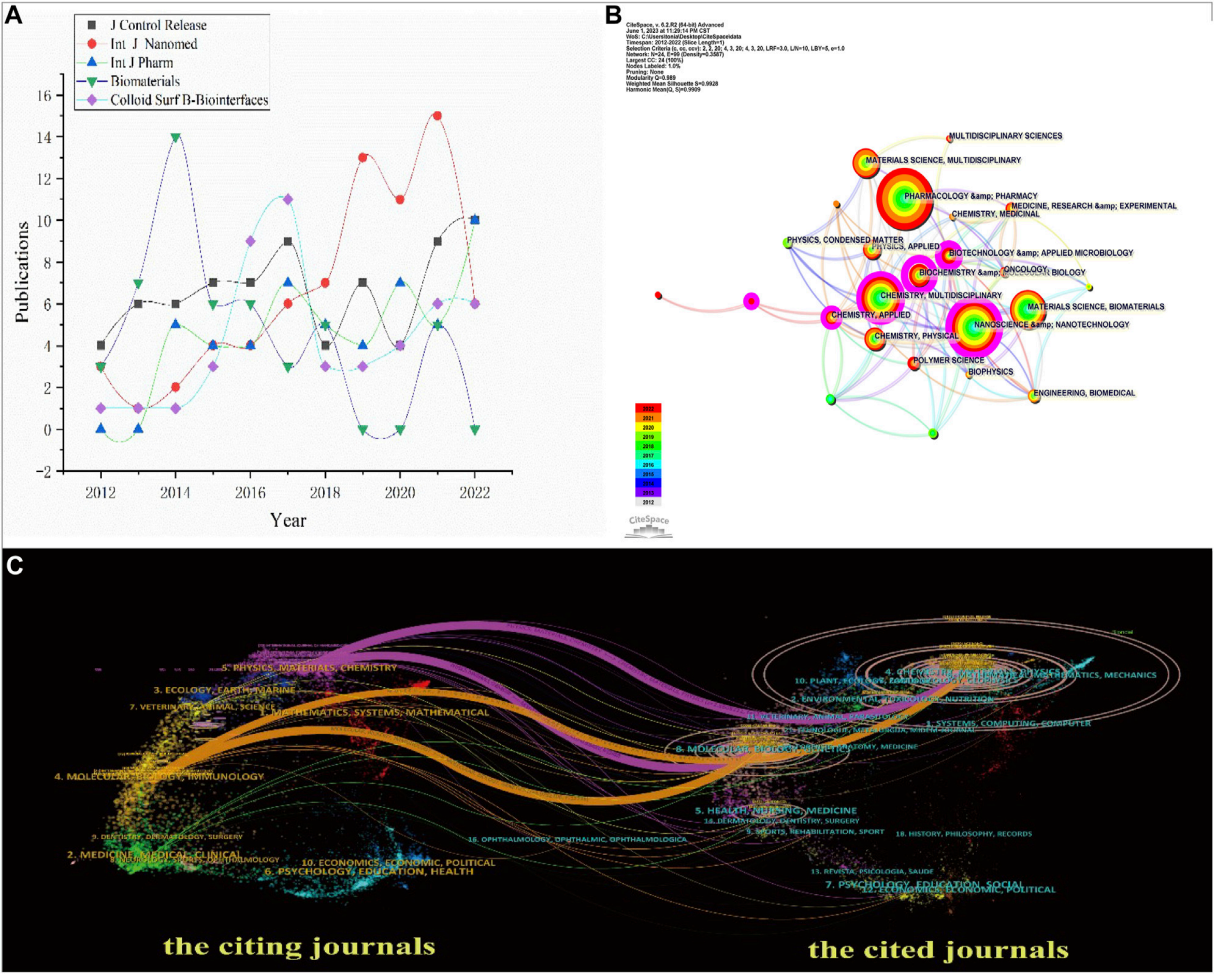
**(A)** Trends of publications ranked in the top five journals 2012–2022. **(B)** Co-occurring subject categories network of NDDSs in BC research. **(C)** Dual-map overlay of journals reflected NDDSs in BC research 2012–2022.

The annual publication counted distribution of the top five journals from 2012 to 2022 is shown in Figure 6A, which shows that the data of all journals did not grow steadily; the *Journal of Controlled Release* had the highest annual growth rate, followed by the *International Journal of Pharmaceutics*. Others evidently fluctuated.

The co-occurring subject categories are shown in Figure 6B. The top three subject categories ranked by quantity are biochemistry and molecular biology, nanoscience and nanotechnology, and biotechnology and applied microbiology.

As shown in Figure 6C, the dual-map overlay of journals shows how the topics in journals were distributed (2012–2022). The labels on the map contain the research topics of all the journals (Chen and Leydesdorff, 2014). The cited journals are on the right; the citing journals are on the map’s left. The distinct coloured lines represented the reference paths, and the colour of a link distinguishes the discipline of the source. To calculate path widths of the connection pathways, the frequency of z-score-scale citation was used. The cited journals were the basis of research. The citing journals were the development and research frontier of the cited literature. All could be classified into 16 clusters, and those cited had ten clusters. Four main citation paths suggested that they are highly correlated on the existing map. We can infer that research commonly published in the Materials/Chemistry/Physics and Molecular/Genetics/Biology preferred to quote published papers in the Materials/Chemistry/Physics or Molecular/Genetics/Biology journals. The research published in the field of Materials/Chemistry/Physics had the most papers.

### 3.6 Analysis of top-yielding co-cited references

From 2012 to 2022, 793 references were co-cited references from the 89,863 references in our investigation. The top-yielding co-cited references, which contain at least 20 articles are shown in Supplementary Table S5A. Bray, F had the most counts; Siegel, R with 67 citations came second. *Ca*: *A Cancer Journal for Clinicians* (IF 2022 = 286.130), *Nature Reviews Materials* (IF 2022 = 76.679), and *Nature Biotechnology* (IF 2022 = 68.164) received the highest IF in the top ten journals.

**FIGURE 7:**
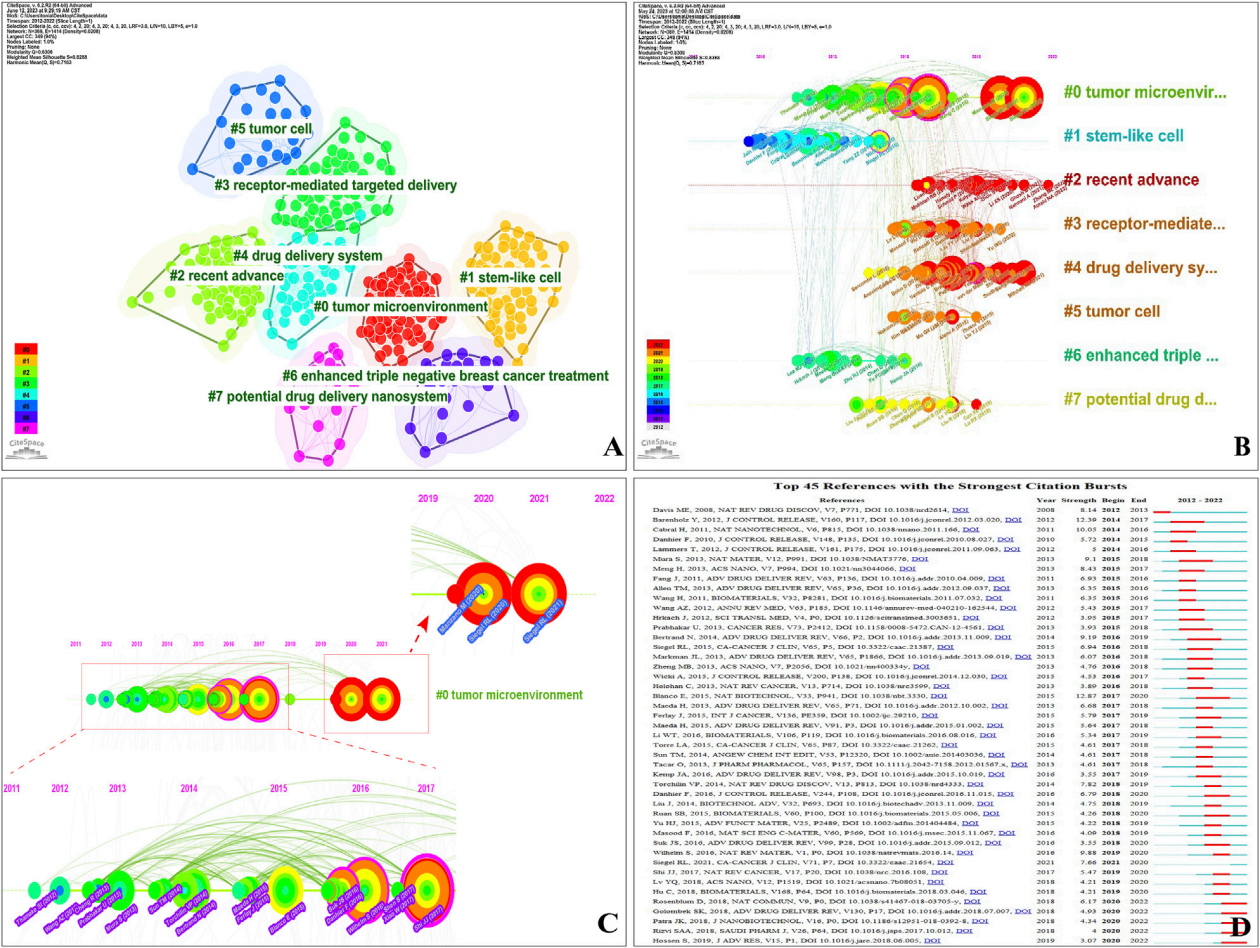
**(A)** Visualization network and cluster of co-cited references. **(B)** Timeline view map of references’ co-cited analysis. **(C)** High-impact members of Cluster #0. **(D)** Top 45 references with citation bursts (sorted by year the burst began). Strength-value denotes the strength of citation bursts; red bars are the time of duration.

To gain a better visualization, we set associated parameters in CiteSpace to show co-citations: the time slice (1 year), the period (2012–2022), the top 10% cited references, and the parameters (N = 369, L = 1,414). Figure 7A represents the network of co-cited references and a visualization cluster map. One circle represents a reference; the same colour represents the same topic.

According to our findings, the modularity Q and the mean silhouette S reached a high of 0.6306 and 0.8288, respectively. This suggests a strong clustering effect and a relatively high level of homogeneity. As shown in Figure 7A, all were classified into eight clusters. Current research directions for NDDSs mainly focus on #0(size, 62) tumour microenvironment (287.11, 1.0E-4), #1(size, 61) stem-like cell (327.06, 1.0E-4), #2 (size, 61) recent advance (229.4, 1.0E-4), #3(size, 57) receptor-mediated targeted delivery (118.68, 1.0E-4), #4(size, 43) drug delivery system (249.01, 1.0E-4), #5 (size, 23) tumour cell (186.43, 1.0E-4), #6(size, 23) enhanced triple-negative breast cancer treatment (182.61, 1.0E-4), and #7(size, 19) potential drug delivery nano-system (149.12, 1.0E-4).

The timeline view visualizes the evolutionary track in topic research, delineating the historical trajectory and time span of references’ co-cited development in each cluster (Cheng et al., 2022a). Each timeline with the most cited references in a particular year is displayed (Chen, 2017). The lines connecting the nodes represent the co-citation relationship. The clusters are placed vertically with sizes in descending order. The coloured curves represent co-citation links added in the year of the corresponding colour. Large-sized nodes or nodes with red tree-rings are used as symbols of citation bursts, highly-cited, or both. A timeline visualization in CiteSpace depicts clusters along horizontal timelines (Figure 7B). Figure 7B indicates the differences in the timeline view with eight clusters (2012–2022). Clusters are labelled 0–7—Cluster #0 is the largest and Cluster #1 is the second largest (Figure 7C). As shown in Figure 7B, some clusters lasted 10 years (until 2022), whereas others were relatively short-lived.

To visually present the evolution and development trend of cited references, we detected the strongest citation bursts between 2012 and 2022 (Figure 7D). The frequency, centrality, and suddenness of cited references could be determined (minimum duration 2). Sorting is by the year the burst began; a slice represents each year. “Strength-value” denotes the citation burst strength. A total of 45 reference bursts were identified. Over time, research hotspots constantly changed: “Davis ME, 2008” occurred earliest (2012); “Hossen S, 2019,” “Rizvi SAA, 2018,” “Patra JK, 2018,” “Golombek SK, 2018,” and “Rosenblum D, 2018” persisted into 2022, demonstrating new fields (Figure 7D). Supplementary Table S5B shows the strongest citation bursts of the top four references.

### 3.7 Keyword analysis

**FIGURE 8:**
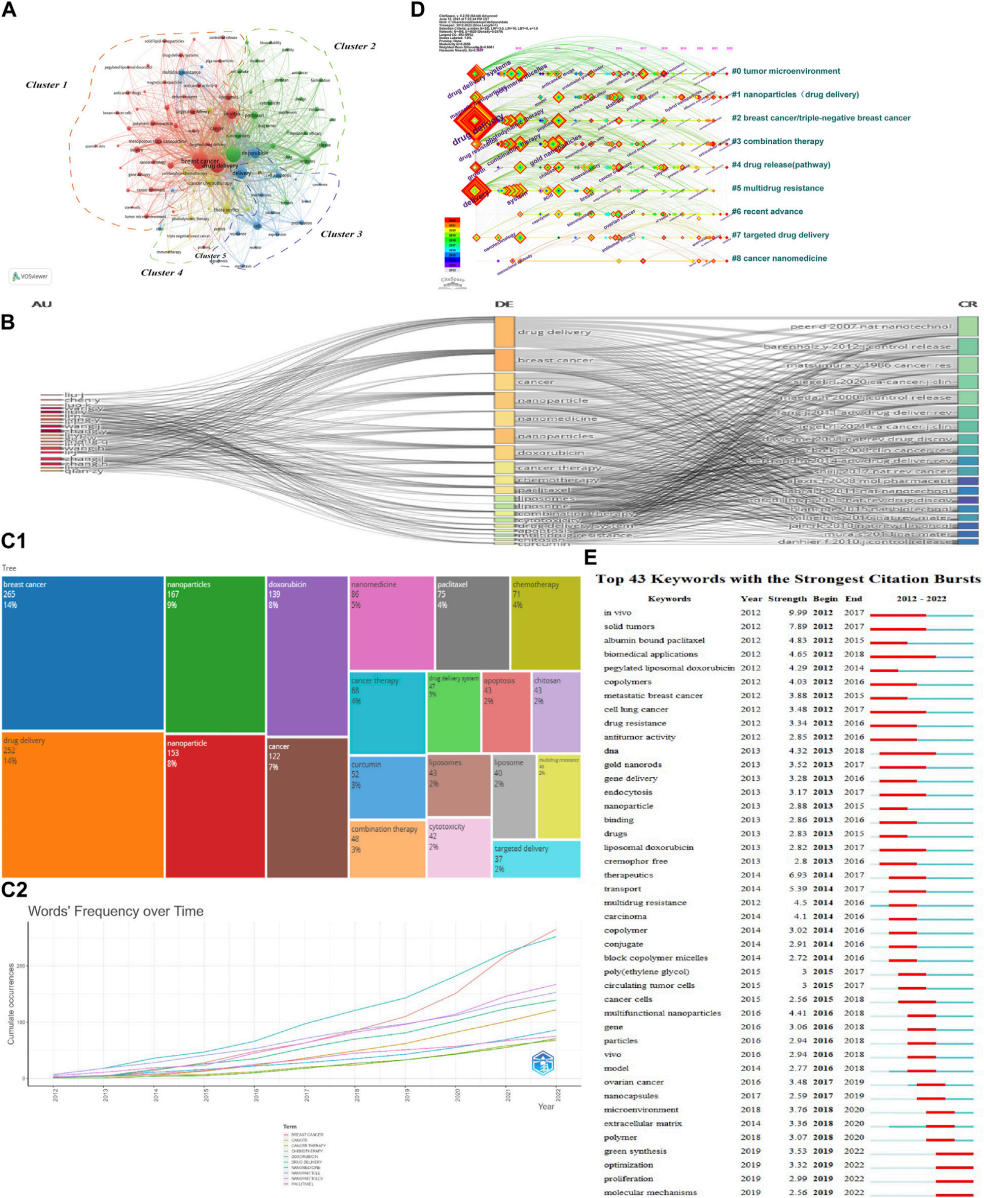
**(A)** Network map of keyword co-occurrence analysis using VOSviewer. Tightly linked keywords were assigned to one cluster of the same colour. Four clusters are listed, shown in red, chartreuse, blue, and yellow nodes. **(B)** Three-field plot showing incoming and outgoing flows among keywords, authors, and references (number of items: 20). **(C1)** Top 20 author keywords of highest frequency. **(C2)** Annual trend of word frequency in author keywords 2012–2022. **(D)** Timeline view map of keyword analysis. **(E)** Top 43 keywords with the strongest citation bursts.

Keywords reflect the core and main content of a paper and also outline its content characteristics. Keywords help us identify the content of interest to the discipline, creating a framework for studies on NDDSs in BC research. Bibliometric tools were used in keyword analysis. First, the tightly linked keywords were assigned to one cluster with the same colour in the keywords co-occurrence network map by VOSviewer (Figure 8A). The 1,752 articles included had 6,925 keywords (author keywords and keywords plus). Some 304 keywords (more than 10 co-occurrence number) met the threshold. Ultimately, keywords with the first 100 were selected. Based on relationship strength and direction, there were five clusters: Cluster 1 (studies on drug delivery and breast cancer); Cluster 2 (studies on nanoparticles); Cluster 3 (studies on delivery and doxorubicin); Cluster 4 (studies on therapeutics and cancer chemotherapy); and Cluster 5 (proteins). Second, the R-tool for performing science mapping analysis is Bibliometrix (Aria and Cuccurullo, 2017). To observe the incoming and outgoing flows of the top 20 keywords, authors, and references contributing to research in the last 11 years, a three-field plot was constructed to represent the network relationship. “Author” was on the left, “Keywords” was in the middle field, and “References” on the right. Figure 8B presents where prolific authors directed their energies (“Keywords”) and the contributions of “References” were massively proportional to each other. From the three-field plot, it was evident that “Zhang L” covered almost all the references that had important keywords like “drug delivery.” Figure 8C1 lists the top 20 terms (“Author Keywords”) with the highest frequencies. The phrase “breast cancer” was first (265), followed by “drug delivery” (252) and “nanoparticles” (167). Figure 8C2 shows the changing trend of annual high-frequency words in “Author Keywords” from 2012 to 2022. Because NDDSs in BC research advanced rapidly, the number of annual global articles rose in the last 5 years. To characterize the dynamic evolution of high-frequency keywords within each cluster, we used a keywords timeline viewer. As shown in Figure 8D, this showed the differences of nine clusters in the appearance time point thus: “Cluster #0 tumour microenvironment,” “Cluster #1 nanoparticles (drug delivery),” “Cluster #2 breast cancer/triple-negative breast cancer,” “Cluster #3 combination therapy,” “Cluster #4 drug release (pathway),” “Cluster #5 multidrug resistance,” “Cluster #6 recent advance,” “Cluster #7 targeted drug delivery,” and “Cluster #8 cancer nanomedicine” (2012–2022). Some clusters remained active until 2022. The crucial sign of this discipline’s evolution is keyword burst strength. To visually present frontiers and hotspots of popular keywords, we detected those with the strongest citation bursts from 2012 to 2022. Over time, keyword frequency, centrality, and suddenness could be determined (minimum duration: 3). A total of 43 keyword bursts were identified (Figure 8E). Among them, “biomedical applications” (2012–2018) received the longest time attention. “*In vivo*” (burst strength 9.99) and “solid tumours” (burst strength 7.89) had the strongest burst strength. “Green synthesis” (2019–2022), “optimization” (2019–2022), “proliferation” (2019–2022), and “molecular mechanisms” (2019–2022) have persisted into 2023, demonstrating new study fields and rising trends in keywords (Figure 8E).

Figure 8E shows the top 43 keywords with the strongest citation bursts. The timeline was described by a blue line, and the red bars indicate the burst period, including start and end years, and the duration of keyword burst.

## 4 Discussion

Notable progress has been made in the application of nanotechnology in the treatment of cancer, especially DDSs (Farjadian et al., 2019). In this study, we focused on the application value of NDDSs in the treatment of BC.

### 4.1 Answers to questions

Q1: What global developmental trends in research have occurred in the field of NDDS-based BC treatment?

As observed previously, curves exhibited a growth trend in annual publications over the past 11 years. Specifically, Figures 2A,B illustrate roughly three stages in publication: acceleration growth (2012–2016), fluctuating (2017–2019), and rapid growth stages (2020–2022). In the first stage, the number of publications and citing articles related to research grew rapidly. This was related to rising research enthusiasm for nanomaterials. In the second stage, worldwide papers on NDDSs in BC research grew slowly and the trend was unstable. Meanwhile, growth in citing articles (annual) began to decline in 2017. This was immediately attributed to the slow progress of drug delivery research. The clinical translation of nanomedicines was greatly hampered. To date, 15 passive targeting nanocarriers (NCs) have been approved for clinical use, and none of the active targeting nanocarriers have made advances in clinical trials (Rosenblum et al., 2018). Clinical research requires the public’s patience to demonstrate safety and efficacy (Park et al., 2022). In the rapid growth stage, publications changed trend and continued to rise substantially. Owing to the progression of COVID-19 mRNA-based vaccines delivered via lipid nanoparticles, researchers renewed their research on NDDSs in the treatment of BC (Kanekiyo et al., 2019; Ramachandran et al., 2022). There will be more citing articles (total) in the near future.

Q2: Which countries/regions, institutions, and authors have been most productive in the field to date, based on scientific collaboration networks?

As is evident from Figure 3A and Supplementary Table S1, the United States and China had more papers on this subject than other countries/regions. China had much more SCP, MCP, and total citations than the United States (Supplementary Table S1). However, the United States still dominates this field with the highest TLS (Figure 3C). In addition, the United States had 47 papers—twice as many as China in 2012. Furthermore, Mylotarg^®^, Doxil^®^, and Abraxane^®^ (albumin–paclitaxel complex approved in 2005) from the United States have become the representative nanomedicines (Park et al., 2022). However, Figure 3D shows that international collaboration was not strong enough in the top ten countries/regions. Therefore, international exchange and cooperation should be strengthened.

Further research is still needed ro examine the contribution of institutions. According to the distribution of institutions’ affiliated countries, half were in China. Universities were the backbone of scientific research. The first institution was Sichuan University. This is partly because it has the National Engineering Research Center for Biomaterials, which has a high academic influence on cancer research in NDDSs and has published several high-quality papers (She et al., 2013; Li et al., 2014). The CAS Key Laboratory for the Biomedical Effects of Nanomaterials and Nano-safety is within the National Center for Nanoscience and Technology of China (Chinese Academy of Sciences, Beijing, China). It has also published much scientific research on nanomaterials targeting treatment of BC. Chinese institutions were core to the research scope of eight clusters (Figure 4B). However, there is much room to improve cooperation and exchange between research institutions from different nations.

Zhang, Yu (Chinese Academy of Science) had the most publications and the highest author contribution rate but did not dominate the field. Their influence index was not high because they had a lower H-index (21) and TLS (17) (Zhang et al., 2016; Zhang et al., 2018b). With a high H-index and the second highest number of publications, Zhang Q (from the School of Pharmaceutical Science, China) dominated this field (Supplementary Table S3) and occupied critical locations in the network map. Zhang Q and team (Dai WB, and Wang XQ) published articles in prestigious scientific journals such as *Biomaterials* (Q1) (Li et al., 2012; Cai et al., 2014), the *Journal of Controlled Release* (Q1) (Mei et al., 2020), *Journal of Materials Chemistry B* (Q1) (Shao et al., 2016), and *ACS Applied Materials & Interfaces* (Q1) (Zhang et al., 2018a).“Redox-sensitive micelles self-assembled from amphiphilic hyaluronic acid–deoxycholic acid conjugates for targeted intracellular delivery of paclitaxel (PTX)” was published by *Biomaterials* in 2012; according to the citation report of WOSCC, it has been cited 390 times. In this article, the main research conclusion showed that a targeted intracellular delivery system of PTX, a promising targeted intracellular delivery carrier, was successfully developed (Li et al., 2012). Looking at the whole picture, Figure 5B shows four groups but weak cooperation. This indicates that academic collaboration among authors was very important in this field.

Q3: What are the top 10 most highly cited papers, the most preferred journals, and the top-yielding co-cited references in the field? What have been the main research directions in the field based on co-citation analysis, and how have they changed over time?

Highly cited papers, top-yielding co-cited references, and the most preferred journals could help us understand current trends. Supplementary Table S4A shows that the paper “Nanomedicine in cancer therapy: challenges, opportunities, and clinical applications,” published in the *Journal of Controlled Release*, was the most popular and highly cited paper on research on NDDSs in BC treatment. The *Journal of Controlled Release* had the most publications (Supplementary Table S4B). These findings suggest that this journal has significantly advanced this topic. With a large number of highly cited papers, it is reasonable to expect a high impact factor for this journal in the coming years. In addition, the related research was mainly in the biochemistry and molecular biology domains by the dual-map overlay of journals.

As can be seen from the timeline view map and the cluster of references in the co-cited analysis, we obtained the topic in the field and predicted research prospects and future cutting-edge research (Chen, 2017). As shown in Figures 7B,C, current research directions for NDDSs mainly focus on “tumour microenvironment,” “stem-like cell,” and “recent advance.” In addition, the burst paper of the most substantial references was “Principles of nanoparticle design for overcoming biological barriers to drug delivery” by Blanco et al. (2015), published in *Nature Biotechnology* (JCR Q1, IF 68.164). In this review, the authors identified biological barriers and proposed principles of NPs design for an efficient delivery system to overcome biological barriers. Its burst began in 2017 and ended in 2020 (Figure 7D). Therefore, overcoming biological barriers via an efficient delivery system is necessary. Alimoradi et al. (2018) developed a stable nitric oxide (NO)-releasing nanoparticle (polystyrene-maleic acid [SMA]-tert-dodecane S-nitrosothiol [tDodSNO]) to enhance the anticancer properties of doxorubicin (Dox) and overcome the biologic barriers of *in vitro* and *in vivo* studies. As delivery systems and nanomedicine platforms, the dual-functionalized graphene oxide (GO)-based nanocarrier, the *Salmonella enterica* serovar Typhimurium, multifunctional hybrid NPs, PEG-PLGA NPs coated with hyaluronic acid, and biomimetic nanoscale erythrocyte could overcome biological barriers to enhance tumour responses to chemotherapy in BC treatment (Suh et al., 2019; Cristofolini et al., 2020; Almoustafa et al., 2021; Liu et al., 2021b; Zhang et al., 2021b).

How have they changed over time? As Figure 7B shows, the research focuses is currently “#0 tumour microenvironment,” “#2 recent advance,” “#3 receptor-mediated targeted delivery,” and “#5 tumour cell.” “#0 tumour microenvironment” contained 62 references over an 11-year period. As is evident from Figure 7C, the timeline visualization revealed three periods of development. The first was 2011–2017 and was full of high impact contributions–large citation tree-rings; periods of citation bursts are coloured red. Several types of high impact contributions appeared in this period. The second period was 2018–2020, which was relatively uneventful and without high-profile references. The third period was 2021–2022 when several types of impactful papers appeared (Siegel et al., 2020; Siegel et al., 2021). Conventional tumour-targeted NDDSs are challenged by the difficulties of drugs released in the tumour microenvironment (TME) and the low efficiency of NPs (Cheng et al., 2020; He et al., 2020).

Q4: What are the main research directions and hotspots of keyword analysis?

Research hotspots are the focus of current research: they are a group of papers with internal connection and a relatively large number in a certain period of time. In bibliometrics, keywords with high frequency and centrality are generally hotspots (Chen, 2006; Song et al., 2022). From the visualization of keywords–cluster analysis, we identified the research directions as Cluster 1 (studies on drug delivery and breast cancer—22 items) and Cluster 2 (studies on nanoparticles—18 items) (Figure 8A). Another crucial sign of rising study trends and hotspots is the timeline view of keywords (Figure 8D). CiteSpace mostly covered the following topics: “tumour microenvironment,” “nanoparticles (drug delivery),” “breast cancer/triple-negative breast cancer,” “combination therapy,” “drug release (pathway),” “multidrug resistance,” “recent advance,” “targeted drug delivery,” and “cancer nanomedicine.” In addition, the strongest keyword bursts were: “*in vivo*” and “solid tumours” (Figure 8E) because NDDSs could offer potential tools for chemotherapeutics for solid tumours.

Q5: What cutting-edge research will develop in the near future?

Citation burst analysis reflects the research directions and changes of the frontier (Xu et al., 2022). The keywords “bursts” and “references,” detected by CiteSpace are crucial signs of cutting-edge research and rising trends (Cheng et al., 2022a). As shown in Figure 8E, the majority of keywords (“green synthesis,” “optimization,” “proliferation,” and “molecular mechanisms”) were related to recent progress and those bursts until 2022. As mentioned previously, these reference bursts persisted into 2022, reflecting the research directions and changes of the frontier. Hossen S, 2019 (strength 3.07), Rizvi SAA, 2018 (strength 4), Patra JK, 2018 (strength 4.34), Golombek SK, 2018 (strength 4.93), and Rosenblum D, 2018 (strength 6.17) had the great potential to be research frontiers. As shown in Supplementary Figure S2, the landscape view generated was based on 1,752 publications from 2012 to 2022.

### 4.2 Core themes of research

#### 4.2.1 Tumour microenvironment

Great progress has been made over the last decade in our knowledge of TME. In comparison with normal tissue, the TME of solid tumours possesses several other particularities, such as hypoxia, acidic pH, reactive oxygen species (ROS), elevated ATP, excessive Zn^2+^, a high level of glutathione (GSH), and higher levels of certain enzymes (Du et al., 2015; Pei et al., 2022). Owing to the increased rate of lactic acid production, the TME of solid tumours is more acidic than that of normal tissue (Webb et al., 2011). Moreover, the aberrant vascular network of tumours disrupts sufficient blood supply to all cells in the tumour mass, leading to hypoxia (Brown and Wilson, 2004). Aerobic metabolism leads to the production of ROS, which are generated by the mitochondrial respiratory chain. ROS levels are substantially higher in diseased than in normal tissues (Pei et al., 2022). In addition, there is a significantly higher amount of GSH in malignant tumour cells, leading to frequencies 1,000 times higher than that in healthy cells (Yin et al., 2018). Furthermore, enzymes such as matrix metalloproteinases are upregulated in TME. These enzymes not only promote cancer cell invasion and metastasis by clearing the pathways of invading cancer cells but also provide nutrients for vascular tumours (Overall and Kleifeld, 2006; Radisky et al., 2017). In recent decades, substantial advances in the development of TME-responsive therapy have offered promising diagnostic and therapeutic strategies. They can exhibit dramatic changes in developing TME-responsive nanomaterials such as pH, temperature, light, reduction/oxidation, and certain enzymes (Uthaman et al., 2018; Wang et al., 2021b).

**FIGURE 9:**
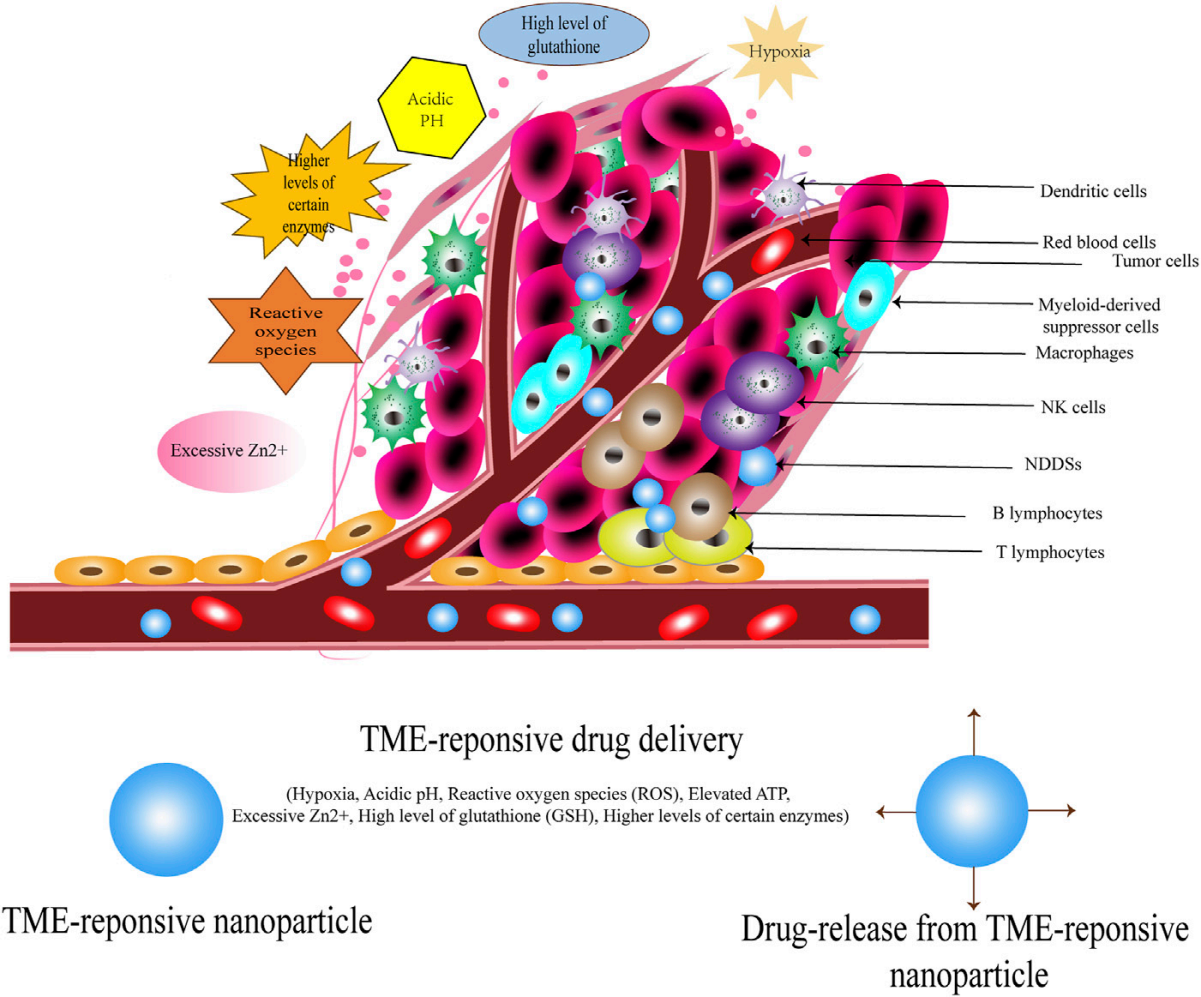
Summary of tumour microenvironment (TME) of solid tumours and TME-related targets. TME of solid tumours possesses several particularities: hypoxia, acidic pH, reactive oxygen species (ROS), elevated ATP, excessive Zn^2+^, and a high level of glutathione (GSH). The researchers designed a series of NDDSs based on these particularities.

The actual anti-tumour effects of nanotherapeutics are limited by various TME factors, such as tumour hypoxia, heterogeneity, and endosomal escape (Uthaman et al., 2018; Hu et al., 2021). Cheng et al. (2022b) developed an NDDS to achieve TME-responsive and targeted delivery of DTA-encoded plasmids (pDTA) to tumour sites via dual targeting to clusters of differentiation-44 and α_v_β_3_ receptors. They subsequently used a combination of losartan and the DTA expression light-switchable transgene system to treat BC based on the NDDS. *In vivo* studies have demonstrated a decrease in active transforming growth factor-β and collagen type I, deeper tumour penetration, and increased survival rate after the novel NDDS application. Abumanhal-Masarweh et al. (2019) reported that treating BC with liposomal bicarbonate combined with a sub-therapeutic dose of Dox achieved excellent therapeutic outcomes, compared with Dox or bicarbonate monotherapy in mouse models of BC. In addition, 100-nm liposomes loaded with sodium bicarbonate were used as adjuvants to elevate TME pH. Liu et al. (2022) administered silibinin and curcumin co-loaded nanoparticles into 4T1 tumour-bearing mice and received an excellent response by inhibiting BC metastasis. Zhang et al. (2021a) designed a novel synergistic cascade strategy (SCS) that involved the use of mild hyperthermia and a smart drug delivery system (SDDS) to alter TME resistance for the effective therapy of TNBC. Shen et al. (2018) engineered, formulated, and delivered genes encoding an IL-10 protein trap to change immunosuppressive TME. This protein trap effectively inhibited the metastasis of TNBC, as shown in Figure 9’s summary of TME of solid tumours and TME-related targets.

As well as their specific targeted effect, the representation of TME in tumour models can lay the foundation for directing combinatorial treatment strategies and cancer nanotherapeutics. Sethi et al. (2015) developed a novel 3D co-culture spheroid model (3D TNBC) representing the tumour milieu of TNBC *in vitro*. Ozcelikkale et al. (2017) developed the TME-on-chip and assessed the delivery and efficacy of Dox in small molecular form *versus* hyaluronic acid-based nanoparticle (NP) formulation in MCF-7 and MDA-MB-231. Two cell lines were representative of different molecular subtypes of BC.

At present, research on TME-related targets is expanding. Researchers have designed a series of NDDSs based on TME factors and TME-related targets (e.g., tumour-associated macrophages (TAMs) and fibroblast activation protein on cancer-associated fibroblasts), which can be easily functionalised for specific targeting (Ji et al., 2016; Cheng et al., 2021). TAM-directed radiotracers and iron oxide NPs are promising tools for targeting TME and for monitoring the effects of immunotherapy via PET and MRI (Mukherjee et al., 2019).

#### 4.2.2 Nanoparticles

**FIGURE 10:**
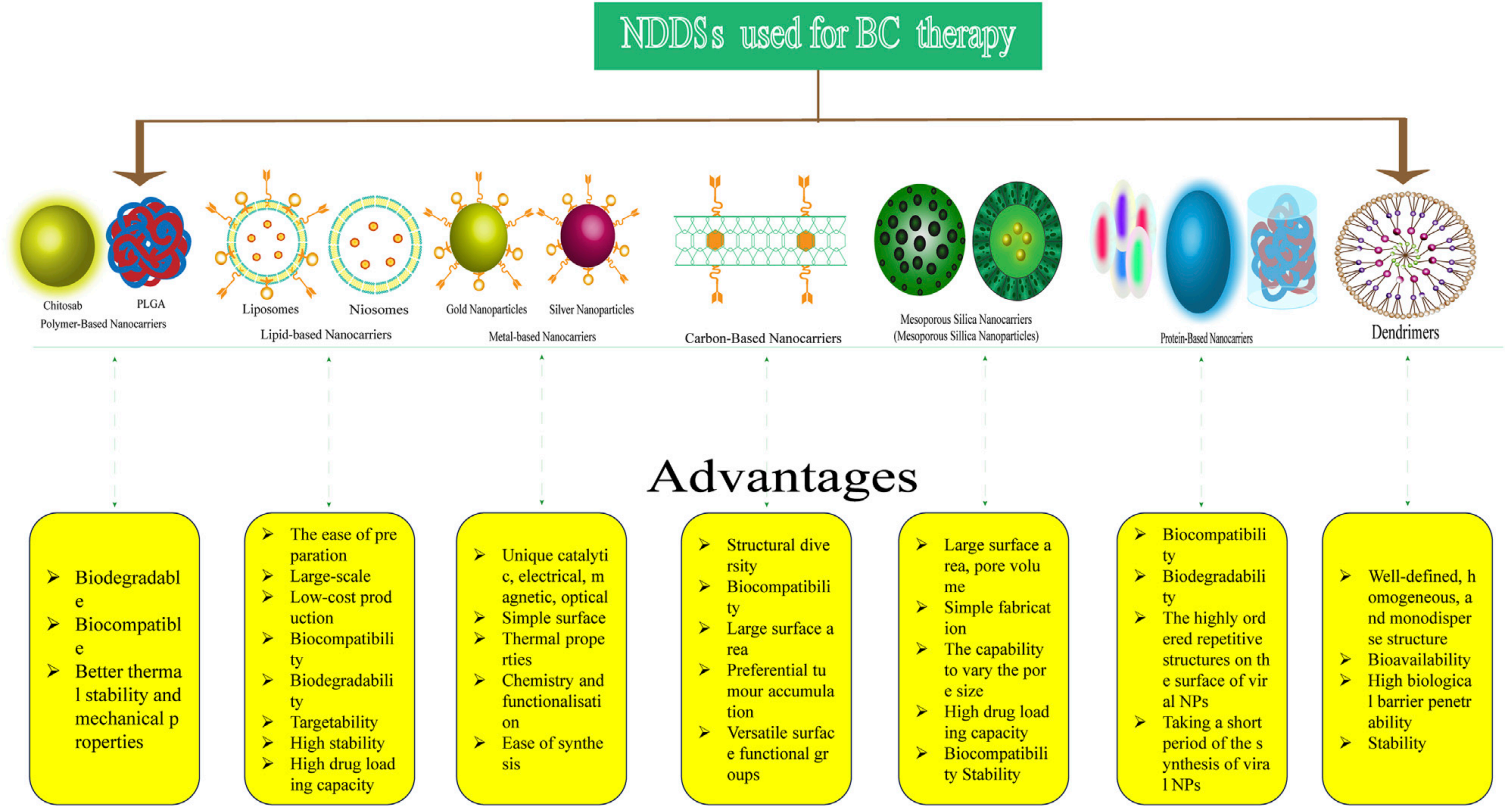
Types of nanoparticle-based drug delivery systems (NDDSs) used for BC therapy and their advantages.

The application of NDDSs to the treatment of cancer has recently gained interest (Liyanage et al., 2019). NPs can deliver drugs more selectively for the treatment of both primary and metastatic cancers. They can help overcome drug resistance, reduce side effects, enhance the bioavailability of poorly water-soluble drugs, and improve drug efficacy (Yap et al., 2021). NDDSs target cancer cells through “passive” and “active” mechanisms. In passive targeting, NPs without targeting ligands accumulate in the tumour interstitial space via the enhanced permeability and retention (EPR) effect (Attia et al., 2019). Active targeting is achieved by the binding of molecular ligands onto the surface of NPs to specific receptors on the tumour cell membrane, which are overexpressed (Patra et al., 2018). Various types of NDDSs with certain advantages have been used in the treatment of BC (Figure 10). The following sections classify these NDDSs based on the structure of NPs and the application of various materials in nanomedicine. The most prevalent classes of nanomedicines are listed in Supplementary Table S6.

##### 4.2.2.1 Polymer-based NPs

Polymer-based NPs (PNPs) are colloidal particles formulated by the interaction of copolymers and a polymer matrix. Their sizes are a few hundred nanometres. PNPs have various shapes and are biodegradable, biocompatible, and thermoresponsive (Soltantabar et al., 2020). Anticancer drugs can be loaded into PNP-based DDSs and delivered to specific targets. In recent years, PNPs have been used to deliver numerous clinical agents such as PTX, Dox, quercetin, trastuzumab, and cisplatin for treating various cancers, including BC (Soltantabar et al., 2020; Giri et al., 2023). In a Phase III clinical trial on the efficacy and toxicity of Genexol-PM and PTX, the drugs were evaluated by Park et al. (2017). Genexol-PM is a freeze-dried polymer micellar preparation of PTX that has low molecular weight, non-toxicity, and biodegradablity. It demonstrated superior clinical efficacy and manageable toxicity compared to conventional PTX (objective response rate of 39.1% vs. 24.3%) in patients with recurrent or metastatic HER2-negative BC. Jin et al. (2018) recently reported the use of photodynamic therapy based on conjugated PNPs for TNBC treatment. They synthesized cyclic arginine–glycine–aspartic acid peptide-decorated conjugated polymer NPs with poly [2-methoxy-5-(2-ethyl-hexyloxy)-1,4-phenylenevinylene] as the photosensitiser for cancer theranostic applications.

However, the side effects of PNPs have been examined via toxicity evaluation and risk assessment. Polymers are mainly classified as synthetic and natural. PNPs are usually considered biodegradable and safe for use. In a study on natural polymers, dextran-coated cellulose-acetate phthalate NPs loaded with 5-fluorouracil were found to have lower toxicity in MCF-7 cells (Singla et al., 2019).

##### 4.2.2.2 Liposomal NPs

From the very beginning of the use of pharmaceutical nanocarriers, the utilization of lipid-based vesicles for drug delivery has been well-established. They are easily prepared and have large-scale and low-cost production, biocompatibility, biodegradability, targetability, high stability, and high drug loading capacity (Rommasi and Esfandiari, 2021). Liposomes are the first generation of lipid-based nanocarriers developed for drug delivery (Yingchoncharoen et al., 2016). They are spherical lipid vesicles with a size range of a few hundred nanometres and are composed of an aqueous core surrounded by at least one phospholipid bilayer. PEGylated liposomal Dox was the first nanomedicine approved by the FDA in 1995 (Barenholz, 2012). Since then, the development of liposomal nano-formulations has accelerated. A randomized open-label phase III study showed that the addition of intrathecal liposomal cytarabine to systemic treatment improved leptomeningeal metastasis (a common manifestation of BC) in the experimental group. The median overall survival of the experimental group was better than that of the control group (OS: 7.3 months [95% CI, 3.9–9.6] *versus* 4.0 months [95% CI, 2.2–6.3]. Median PFS: 3.8 months [95% CI, 2.3–6.8] *versus* 2.2 months [95% CI, 1.3–3.1]) (Le Rhun et al., 2020).

Niosomes, another type of lipid-based nanocarrier, are spherical vesicles consisting of closed bilayer structures that arise from the self-clustering of cholesterol and non-ionic surfactants in aqueous media. Niosomal nano-formulations of Dox, triaryl-(Z)-olefin (TZO), and tamoxifen citrate have higher anticancer efficacy in *in vitro* and *in vivo* studies (Nowroozi et al., 2018; Salem et al., 2018; Salem et al., 2020).

Solid lipid NPs (SLNs) are composed of solid lipid nuclei coated with surfactant layers which are stable in aqueous environments. A study showed that curcumin-loaded SLNs had better anti-tumour effects than free curcumin in BC cells (Wang et al., 2018). Zheng et al. (2019) used Dox-loaded SLNs: an arginine–glycine–aspartic (RGD)-conjugated, pH-sensitive lipid was synthesized using glycerin monostearate (GMS) and adipic acid dihydrazide (HZ) as lipid materials, ultimately named RGD–HZ–GMS. They evaluated the anticancer effect of RGD–Dox–SLNs in a BC cell line (MCF-7) and a Dox-resistant cell line (MCF-7/ADR). The results indicated that RGD–Dox–SLNs exerted anticancer effects and reduced multidrug resistance in BC cells. Furthermore, MCF-7 cells were exposed to liposomes encapsulating hydroxy urea and artemisinin. Preliminary results suggested that hydroxy urea-loaded liposomes (IC_50_ = 43.78 ± 0.017 mg/mL) had higher cytotoxicity than the free drug (IC_50_ = 69.11 ± 0.005 mg/mL) against the MCF-7 cell lines (Talluri et al., 2016).

##### 4.2.2.3 Metal-based NPs

Metal-based NPs (inorganic NPs) are colloidal particles with diameters ranging from 10 to 1,000 nm (Desai et al., 2021). They are characterized by unique catalytic, electrical, magnetic, optical, and thermal properties; simple surface chemistry; and functionalisation and ease of synthesis (Sharma et al., 2018). Metal-based NPs such as gold NPs (AuNPs), superparamagnetic iron oxide NPs (SPIONs), silver NPs (AgNPs), and quantum dots (QDs) have been most widely used in the treatment of BC.

###### 4.2.2.3.1 Gold NPs

NPs can retain their form owing to their resistance to chemical oxidation renders and have been widely applied in biomedicine. The most common synthesis of AuNPs involves the Au^3+^ reduction by citrate in aqueous media. The drugs or therapeutic agents can be loaded onto AuNPs. They then bind to the surface through covalent or non-covalent bonds and release at the target site (Prabaharan et al., 2009). Jafarizad et al. (2017) evaluated the efficiency of AuNPs and AuNPs/RGO composites for MCF-7 breast cancer cells, with satisfactory results. AuroLase^®^ AuNPs are under investigation in clinical trials (Anselmo and Mitragotri, 2015).

###### 4.2.2.3.2 Superparamagnetic iron oxide NPs

SPIONs are NPs between 10 and 100 nm in size. Their inner core is comprised of magnetite, Fe_3_O_4_, or maghemite *γ*-Fe_2_O_3_. The magnetic core is thus covered by a hydrophilic coating for stabilization, such as polymers targeting delivery to specific sites. Poller et al. (2017) analysed the impact of several characterized SPION types (varying in size, zeta potential, and surface coating) on human BC cell lines to identify the most suitable particle. They found that treatment with dextran-coated SPIONs and lauric acid-coated SPIONs (SPIONLA) with a protein corona formed by human serum albumin (SPIONLA-HSA) could form a very moderate particle uptake and low cytotoxicity. Jeon et al. (2019) developed an NP formulation (NP-PTX-FA) comprising a superparamagnetic iron oxide core coated with short and long chain polyethylene glycol. They found that NP-PTX-FA had strong toxic effects on BC cells.

###### 4.2.2.3.3 Silver NPs

AgNPs ranging from 1–100 nm in size are an important class of nanomaterial for a wide range of industrial and biomedical applications. They have shown promising anticancer effects. Gurunathan et al. (2013) reported that AgNPs inhibited the viability and growth of MDA-MB-231 cells (an epithelial, human breast cancer cell line) and induced membrane leakage in a dose-dependent manner. AgNPs exerted cytotoxic effects by inducing apoptosis, and ROS generated by AgNPs were found to play an important role in apoptosis.

###### 4.2.2.3.4 Quantum dots

QDs, 2–10 nm in size, are semiconductor nanocrystals that contain a metal inner core. They are highly fluorescent and have advanced photophysical and spectral properties, including high brightness and stability against photobleaching. Water-soluble QDs are used for biomedical applications. The conjugation of QDs with surface-modifying ligands and peptides are used in target-specific cancer studies (Bilan et al., 2016). Sun et al. (2018) developed a water-soluble biomarker for detecting BC using a CuInS_2_/ZnS quantum dot-labelled Ki-67 bioprobe. The experimental results indicated that the QD–Ki-67 probes retained the original optical properties of the unadorned QDs and did not exhibit distinct toxic side effects *in vitro* cytotoxicity experiments. There is currently one clinical trial of QDs. CdS/ZnS core–shell-type QDs coated with veldoreotide have been developed for the suppression and bioimaging of BC (Ph I). During the protocol, female BC patients received either FDA-approved local medication as a negative control drug or peculiar therapeutic where CdS/ZnS core–shell-type QDs coated with veldoreotide in the form of topical cream get deposited deep in the breast periphery as an anticancer drug (Huang et al., 2020).

##### 4.2.2.4 Carbon-based NPs

There are three naturally existing carbon allotropes and several synthetic carbon allotropes, including carbon nanotubes (CNTs), carbon nanocones, carbon nanohorns, fullerene, graphene, and nanodiamond (Yap et al., 2021). Carbon-based NPs have unique properties such as their small size, highly specific surface area, benign biocompatibility, low-toxicity, and versatile surface functional groups. They have been widely used as drug carriers in medical applications (Yan et al., 2016). CNTs are allotropes of fullerene with cylindrically shaped long, hollow structures with a wall composed of graphene sheeting rolled at a specific angle. Neves et al. (2013) reported the targeting of single-walled carbon nanotubes (SWNTs) for treating breast cancer with minimal side effects using photothermal therapy. Garriga et al. (2020) investigated the *in vitro* toxicity of carbon nanomaterials in MCF-7 cells, such as carbon nanohorns (CNH), CNTs, carbon nanoplatelets (CNPs), GO, reduced GO (RGO), and nanodiamonds (NDs). They found cell viability after carbon nanomaterial treatment followed the order CNP < CNH < RGO < CNT < GO < ND. CNP produced remarkably high ROS levels.

##### 4.2.2.5 Mesoporous silica NPs

Mesoporous silica NPs (MSNs) have unique properties such as large surface area and pore volume. Their pore size can be modulated to specific requirements. Owing to these characteristics, MSNs deliver drugs without premature release before reaching the target site. MSNs are good carriers of easily degradable molecules such as genes and proteins. Zhao et al. (2018) developed a new pH-sensitive NDDS composed of MSNs. Compared with the conventional approach, the MSNs exhibited a tenfold increase in killing ability. The enhanced effects of the new pH-sensitive NDDS on MDR reversal were attributed to its higher uptake rate in MCF-7 cells. *In vivo* experiments demonstrated that the novel NDDS demonstrated better efficacy against multidrug-resistant tumours in mice and targeted the tumour site more effectively with minimal toxicity.

##### 4.2.2.6 Protein-based NPs

Protein-based NPs (viral NPs) are a group of NPs resembling the protein envelopes or capsids of viruses. Viral NPs are emerging as a versatile tool for targeted drug delivery with the advantage of the highly ordered repetitive structures on the surface of viral NPs; they also synthesize more quickly (Liyanage et al., 2019). Esfandiari et al. (2016) proposed that viral NPs could be produced inexpensively on a large scale. Meanwhile, the team used NPs formed from the potato virus X to conjugate Herceptin (trastuzumab) monoclonal antibody: a new option in specifically targeting breast cancer.

##### 4.2.2.7 Dendrimers

Dendrimers have three-dimensional polymeric macromolecules composed of a typically symmetric core, an inner shell, and an outer shell. They are nano-sized, radially symmetric molecules with a well-defined, homogeneous, and monodisperse structure (Abbasi et al., 2014). Owing to their characteristics (polyvalency, self-assembly, electrostatic interactions, chemical stability, low cytotoxicity, and solubility), various types of dendrimers have been extensively used for biomedical applications. They are considered ideal NDDSs for the treatment of BC because they enhance the solubility, dissolution, adsorption, bioavailability, stability, and efficacy of the loaded drugs and facilitate targeted drug delivery (Abbasi et al., 2014; Sherje et al., 2018; Dubey et al., 2021).

The new NPs, such as exosomes, human umbilical cord-derived mesenchymal stem cells (huc-MSCs), have been engineered to function as NDDSs of chemotherapeutic drugs against BC (Cao et al., 2018; Al-Humaidi et al., 2021). Nano-formulations of medicinal plant extracts/essential oils and bioactive compounds will be of great importance for BC research (Lahiani et al., 2017; Majidzadeh et al., 2020; Yap et al., 2021). In addition, thermo-sensitive nanocarriers combined with photothermal therapy and targeted chemotherapy, temperature-sensitive response systems, and NDDSs that target specific proteins expressed on the tumour cell membrane are other therapeutic strategies for improving the treatment effect of BC (Dorjsuren et al., 2020).

#### 4.2.3 Breast cancer and triple-negative BC

**FIGURE 11:**
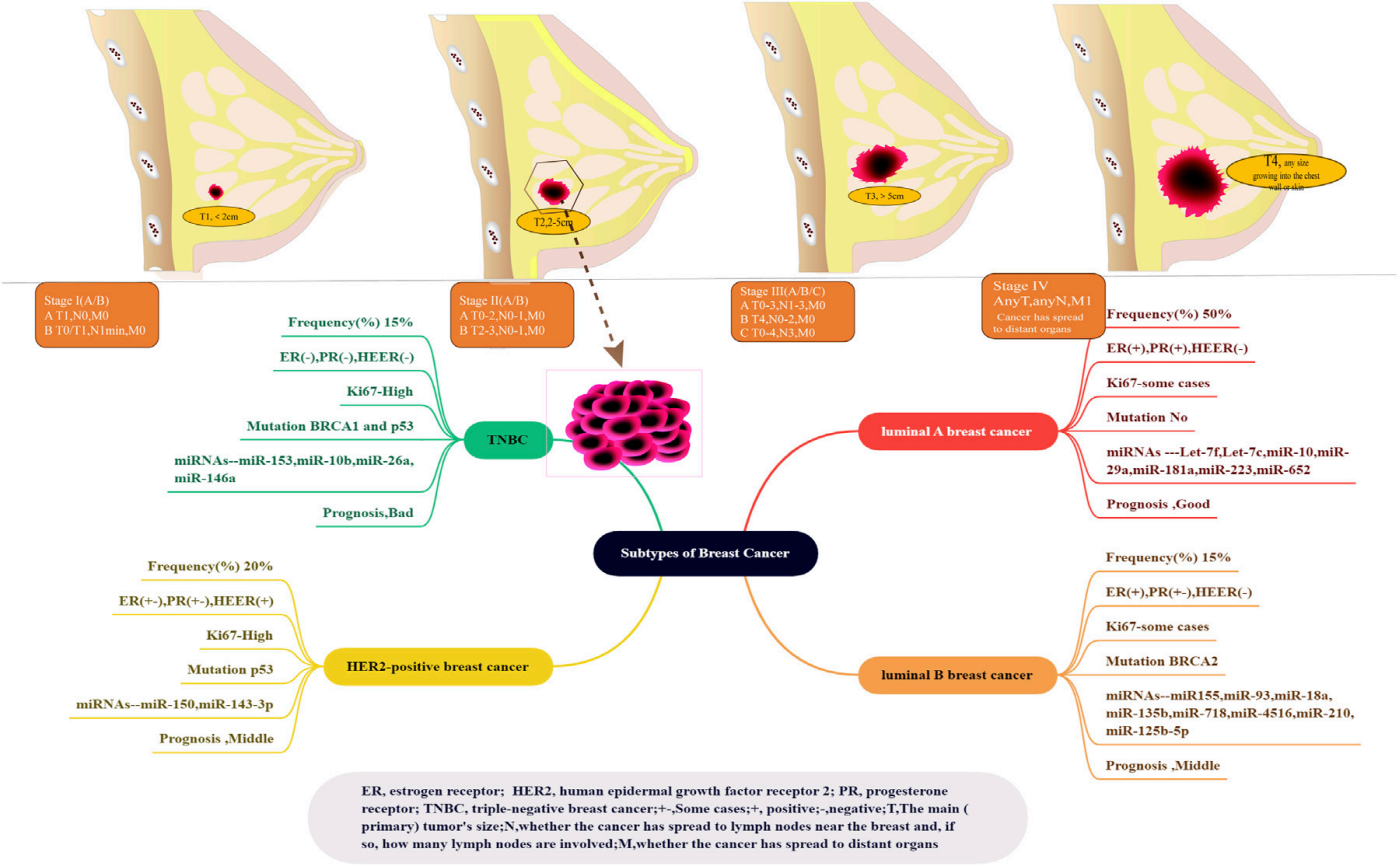
Subtypes of BC and BS stages (Orrantia-Borunda et al., 2022). Top indicates five stages in BC, which is approved by Cancer.Net. The lower part shows another inclusion criteria. BC classification is luminal A, luminal B, HER2-positive, and triple-negative (four subtypes of BC), which are widely recognized based on molecular expression. The current clinical models for the classification of BC enjoy the advantages of several molecular markers, including miRNAs (let-7, miR-155, miR-150, and miR-153) and mutations (p53 and BRCA 1 and 2 genes).

As mentioned previously, BC is projected to be the most common cancer and the second leading cause of cancer-related death among women in 2023 (Siegel et al., 2023). BC is diagnosed based on standardized pathological criteria in clinical settings. Approximately 50%–75% of BC cases are attributed to invasive ductal carcinoma, whereas 5%–15% of cases are attributed to invasive lobular carcinoma (Dillon et al., 2014). The remaining cases are classified under special histological types, such as mixed ductal/lobular carcinomas (Dillon et al., 2014). BC comprises 20 morphologically distinct subclasses, such as cribriform, papillary, spindle cell, solid, neuroendocrine, clinging, medullary, clear cell, secretory, and mucinous (Hsiao et al., 2010). At present, molecular classification is widely accepted, whereas morphological classification is uncommon. Two main molecular targets have been identified: oestrogen receptor alpha (ERα)/steroid hormone progesterone receptor (PR) and epidermal growth factor 2 (ERBB2, HER2, or HER2/neu). ERα is a steroid hormone receptor and transcription factor. It expresses in approximately 70% of invasive BC, activating growth pathways. PR is also a principal marker of ERα signalling (Joshi, 2018). The expression of ER or PR is defined as HR+, when 1% of tumour cells are over-expressed. Additionally, ERBB2, HER2, or HER2/neu is a transmembrane receptor tyrosine kinase in the epidermal growth factor receptor family. It is significantly overexpressed in approximately 20% of BC. The amplification of the gene ERBB2 is defined as ERBB2+ (Wolff et al., 2007). TNBC is characterized by a lack of expression of ER, HER2, or PR and accounts for approximately 15% of BC cases (Denkert et al., 2017). These three molecular subtypes of BC have distinct prevalence rates and prognoses and require different systemic treatments. According to TNM classification, BC is divided into four stages. Stage I is the presence of breast tumours of <2 cm in size without lymph node involvement, whereas Stage IV is defined by metastasis from the breast and axilla to distant sites—most commonly bones, brain, liver, and lungs (Chavez-MacGregor et al., 2017) (Figure 11). Stage IV BC accounts for approximately 6% of all BC cases in the United States (Lee et al., 2020).

#### 4.2.4 Chemotherapy associated with BC treatment

**FIGURE 12:**
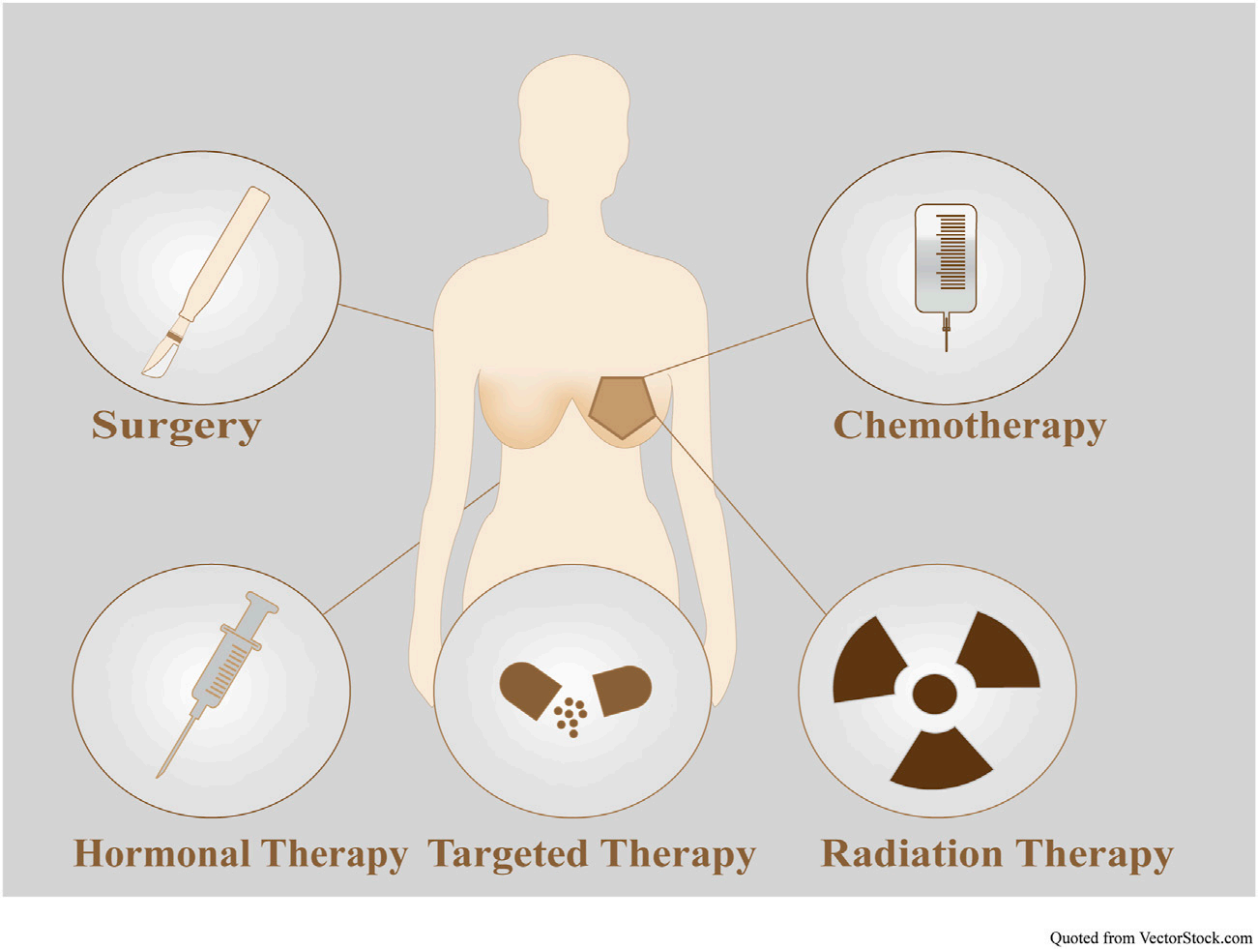
Combination therapies for BC. To date, BC treatment includes surgery, chemotherapy, immunotherapy, endocrine therapy, targeted therapy, and radiation therapy.

The progress of BC treatment is not limited to surgery, chemotherapy, immunotherapy, endocrine therapy, targeted therapy, and radiation therapy (Figure 12; Supplementary Table S7). Eradicating regional lymph nodes and breast tumours to prevent metastasis and recurrence is the primary principle of BC treatment. Local therapy includes surgical resection, axillary lymph node sampling, or removal, followed by postoperative radiation. BC therapy can be preoperative or postoperative. Different systemic therapies are required for treating different BC subtypes, such as endocrine therapy for HR+ BC, trastuzumab-based antibody therapy, and chemotherapy for all ERBB2+ tumours and chemotherapy alone for TNBC. Maintaining basic survival and improving patient quality of life are primary approaches to treating metastatic BC. Adjuvant approaches are common between metastatic and nonmetastatic BC. Surgery and radiation are typically used to alleviate symptoms in metastatic BC (Waks and Winer, 2019).

Chemotherapy essentially includes adjuvant and neoadjuvant therapy in the treatment of BC. In this section, we summarise the significance of randomized clinical trials that investigate the efficacy of adjuvant and neoadjuvant chemotherapy (NAC) for BC (Supplementary Figure S3). NAC is recommended in patients with locally advanced disease or aggressive tumour biology before surgical resection (Leon-Ferre et al., 2021). The advent of adjuvant chemotherapy in BC was by Bonadonna et al. (1976). The first chemotherapeutic regimen prescribed was a combination of cyclophosphamide, methotrexate, and 5-fluorouracil (CMF). CMF was used as an adjuvant treatment to radical mastectomy in patients with primary BC with positive axillary lymph nodes and prevented metastatic recurrence (5.3% of 207 patients treated with chemotherapy vs 24% of 179 patients without chemotherapy). A 1990 study reported that the therapeutic effects of four cycles of Adriamycin and cyclophosphamide (AC4) demonstrated a non-inferiority margin for 6 months of CMF in patients with tamoxifen-nonresponsive positive-node BC (Fisher et al., 1990). A 1999 trial explored the effectiveness of concurrent *versus* sequential regimens and demonstrated that treatment with AC4 followed by four cycles of docetaxel (ACT) improved the survival of patients with invasive adenocarcinoma (tumour stage T1, T2, or T3; clinical nodal stage N0 or N1; and metastasis stage M0). Compared with doxorubicin–docetaxel and the concurrent-ACT group, the result of the trial of the sequential-ACT group was positive (8-year DFS: 74% vs 69% vs 69%, respectively). However, no significant improvement in OS was seen (8-year OS: 83% vs 79% vs 79%, respectively) (Swain et al., 2010). Until 2001, the role of trastuzumab in HER2+ BC was presented, especially for metastatic HER2+ BC. In the trial, trastuzumab plus anthracycline and cyclophosphamide (AC) vs AC-alone was used. The result demonstrated the therapeutic effects by progressive disease (7.4 months vs 4.6 months; *p* < 0.001), objective response (50% vs 32%, *p* < 0.001), duration of response (median, 9.1 vs 6.1 months; *p* < 0.001), and longer survival (median survival, 25.1 vs 20.3 months; *p* = 0.01) (Slamon et al., 2001). In a 7-year follow-up of US oncology research trial 9735, four cycles of docetaxel/cyclophosphamide (TC) were found to have better therapeutic effects than four cycles of an anthracycline (doxorubicin)/cyclophosphamide (AC) in early BC (DFS, 81% vs. 75%, respectively; HR, 0.74; OS, 87% vs. 82%, respectively; HR, 0.69) (Jones et al., 2009). In the 2017 ABC trials, the analysis of TC6 *versus* the TaxAC regimens (various anthracycline-plus-taxane-containing regimens) was planned, with invasive disease-free survival (IDFS) as the primary end-point for early BC. The TaxAC regimens were proven to not be non-inferior to various TC6 (4-year IDFS, 90.7% for TaxAC vs 88.2% for TC6; *p* = 0 .04) (Blum et al., 2017). In addition, the NSABP B-18 trial conducted was the first study to compare the outcomes of preoperative and postoperative AC in 1998. The results suggested that preoperative chemotherapy was as effective as postoperative chemotherapy, and that there was no significant difference in DFS, DDFS, and survival (*p* = 0.99, 0.70, and 0.83, respectively) between both groups. Meanwhile, many women have undergone lumpectomy (Fisher et al., 1998). NAC is the preferred therapeutic approach for TNBC and HER2-positive BC (Leon-Ferre et al., 2021). Chemotherapeutic drugs, including Dox and PTX, have also been used in combination with drugs such as disulfiram, thioridazine, and curcumin to improve therapeutic outcomes (Núñez et al., 2016).

#### 4.2.5 Challenges and multidrug resistance-associated with chemotherapeutic treatment of BC

Despite progress in the existing treatment strategies, the treatment of brain metastases has not advanced in parallel. The treatment of metastatic BC is challenging, partly attributed to the limited blood–brain barrier penetration of anti-neoplastic agents and genetic heterogeneity-activation of the PI3K/AKT/mTOR pathway involved (Dagogo-Jack et al., 2017; Bowman and Kumthekar, 2018). Toxicity associated with chemotherapeutic agents can directly or indirectly cause, for example, anaemia, cardiotoxicity, pulmonary toxicity, nephrotoxicity, and thrombocytopenia.

NDDSs may be modified to improve the treatment of BM in BC. However, efficient drug delivery to the tumour is still unsolved. Biological barriers to drug transport prevent the successful accumulation of chemotherapeutic agents at the lesion location. TME is a barrier to NDDSs since TME of solid tumours possesses particularities like hypoxia and acidic pH (Du et al., 2015; Pei et al., 2022). High interstitial fluid pressure, desmoplastic stroma, and TAMs hinder the diffusion of nanotherapeutics, thereby reducing the cellular uptake rate (Blanco et al., 2015; Cheng et al., 2021). The efficacy of chemotherapeutic drugs administered via NDDSs is severely limited owing to poor and nonselective cellular uptake and unstable circulation; further clinical trials are warranted (Wang et al., 2021c). In addition, NP transport is related to the administration route, which determines the biodistribution pattern of drugs. Oral, intradermal, and subcutaneous routes of administration should be further explored in future studies (Wang et al., 2021a).

The other major obstacle is the development of MDR. It can be categorized as primary (manifesting as tumour insensitivity to initial treatment) or acquired (occurring after initial response to therapy) (Perez, 2009). Chemotherapeutic agents have transient responses because of MDR (Gorain et al., 2018). Tumours may not respond to systemic therapies; in particular, metastatic BC does not respond to initial treatment. Consequently, many studies have used liposomes (Deng et al., 2014), NPs (Pan et al., 2013), micellar systems (Qiu et al., 2014), siRNA-targeted particular genes (Conde et al., 2013), and exosomes (McClements, 2020) to overcome MDR. NDDSs may offer additional benefits by overcoming the limitations of conventional treatment, including difficulties in targeting, dynamic *in vivo* changes in the materials, and multiple biological barriers. However, these experimental results need further testing.

#### 4.2.6 Systemic delivery and biodistribution

**FIGURE 13:**
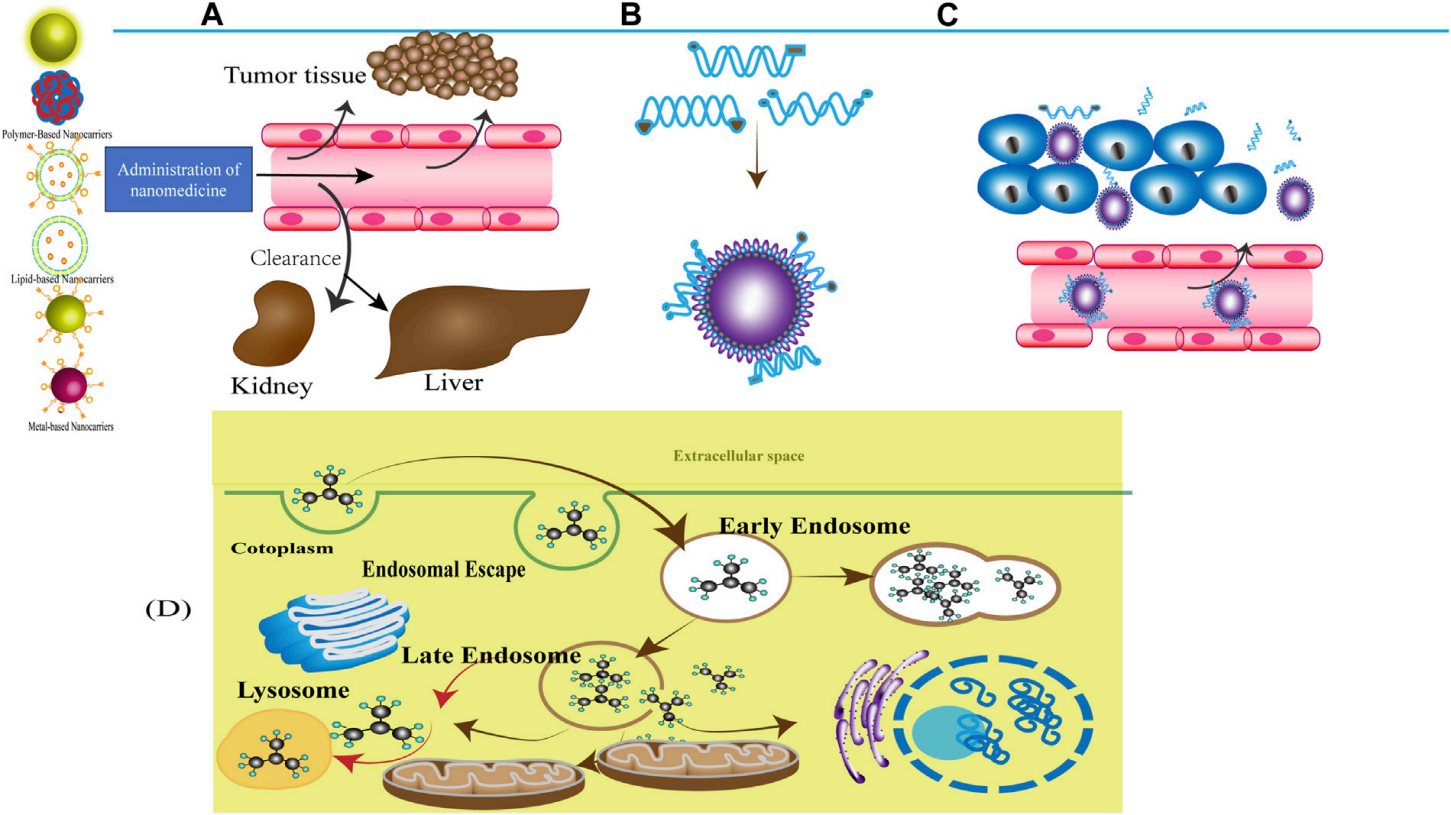
Systemic delivery and biodistribution **(A)** Administration of nanomedicine, NDDSs reach the target position mainly through blood circulation. And the major organs responsible for nanoparticle excretion are the kidneys and the liver. **(B)** NP–protein interactions. The term “protein corona” (PC) is used to describe the layer that proteins form around NPs when placed in a biofluid. Proteins on the NP are always in the dynamic process of adsorption and desorption. With the prolongation of exposure time, the low affinity and high concentration of proteins close to the NP would be replaced by proteins with high affinity and low concentration, which rearrange at the surface of the particle and eventually form an irreversible stably hard corona composed of tightly bound proteins, while the replaced proteins absorbed on the outside form a reversible unsteadily soft corona consisting of loosely bound proteins [172]. **(C)** Blood circulation The process of NPs‘ distribution from the blood circulation to a specific organ/tissue/cell. Vascular extravasation into the TME which can be influenced by the perivascular TME, aberrant tumour vasculature. **(D)** Cellular uptake and intracellular trafficking-Scenarios of Carrier-Mediated Endosomal Escape and Subcellular Delivery of Cargos. Schematic of endocytosis and endosomal escape. Particles entered the cells via the endocytic pathway become entrapped in the visieles, the vesicles matured form early endosomes and late endosomes and eventually end up in the lysosome, the particles are effective by achieving the endosome escape. Alternatively, it is degraded by enzymes in the lysosome.

As shown in Figure 13, enhancing drug delivery to tumours is urgently needed for many steps, including NP–protein interactions, retention of NPs in blood circulation, extravasation of NPs into the TME, penetration of tumours by NPs, cellular uptake and intracellular trafficking of NPs, and controlled drug release.

Adsorption of proteins on the surface of NPs in a biological milieu results in the formation of protein corona. Proteins on the surface of NPs continuously undergo the dynamic process of adsorption and desorption (Cai and Chen, 2019). These changes influence NPs’ surface properties, electric charges, hydrodynamic size, and their other physicochemical processes, including their corrosion and coagulation (Zarei and Aalaie, 2019). In addition, they affect cellular uptake, intracellular trafficking, PK, biodistribution, and toxicity (Shi et al., 2017).

Blood circulation refers to NPs that are distributed to a specific organ/tissue/cell through blood circulation. Therefore, blood circulation half-life—the efficiency with which NPs passively extravasate from the microvasculature into the TME—is an important pharmacokinetic (PK) parameter of NPs. NPs should be retained in blood circulation for a long period to ensure the delivery of adequate concentrations of drugs to target organs/tissues/cells; thus, long-circulating NPs coated with an inert polymeric material should be developed. PEGylated NPs offer a strategy for decreasing immunogenicity and prolonging circulation time, thus overcoming various biological barriers (Suk et al., 2016). PEG is usually incorporated onto the surface of liposomes or inserted after vesicle formation (Ho et al., 2013), limiting the direct interaction between various blood components and liposomes (Takakura and Takahashi, 2022). The other major factor limiting circulation time is the non-specific interaction between NPs and serum proteins (protein corona formation). The composition and quantity of protein corona largely depend on the physicochemical properties of NPs, especially their *in vivo* pharmacokinetic properties (Bertrand et al., 2017). Rodriguez et al. (2013) developed nano-formulations that used minimal “self” peptides attached to virus-size particles. Intravenous injection of this nano-formulation into mice resulted in persistent retention that enhanced dye and drug delivery to tumours.

NPs enter the tumour site from systemic circulation through extravasation, which can be influenced by the perivascular TME and aberrant tumour vasculature. Because of the metabolic demands of rapid cancer cell division, the tumour neovasculature is quite distinct from the normal vasculature and exhibits “leakiness.” Some vascular mediators such as nitric oxide and angiotensin II may enhance the extravasation of NPs from tumour blood vessels (Seki et al., 2009; Matsumoto et al., 2016). Therefore, normalising the tumour vasculature can improve anti-tumour efficacy and improve drug delivery efficiency (Magnussen and Mills, 2021). Iron oxide NPs, cuprous oxide, AuNPs, silica NPs, MAN-PLGA, and APRPG-PEG-ZOL-CLPs have been used to regulate the normalisation of tumour vasculature in BC models (Liang et al., 2022).

Tumour penetration can affect NPs in DDSs, hindering their penetration into the tumour core region and limiting their diffusion. The dense interstitial matrix (Li et al., 2018) and high abundance of perivascular stromal cells (Miller et al., 2015) in the TME impede NP penetration into the tumour site (Shi et al., 2017). Zhang et al. (2020) fabricated small morph NPs (PDMA) by modifying polyamidoamine (PAMAM) dendrimers with dimethylmaleic anhydride (DMA). PDMA facilitates deep penetration through active, energy-dependent caveolae-mediated transcytosis.

Cellular uptake and intracellular trafficking mainly refer to enhancing the retention of NPs at the cellular level through internalisation pathways. For effective intracellular delivery, targeting the nanocarrier to a specific organ/tissue/cell from blood circulation is the first step, after which NPs are internalised by cells via endocytic pathways. In addition, NPs can be optimised to enhance their cellular uptake and achieve specific targeting. NPs can also be optimised to enhance their cellular uptake and achieve specific targeting. For optimal intracellular delivery, NPs should be able to evade degradation, have effective and efficient targeting ability, and facilitate the controlled release of drugs into the intracellular environment. To this end, multiple actively targeted NPs have been developed (Prabaharan et al., 2009; Neves et al., 2013; Bilan et al., 2016; Park et al., 2017; Jin et al., 2018; Nowroozi et al., 2018; Patra et al., 2018; Sherje et al., 2018; Sun et al., 2018; Wang et al., 2018; Soltantabar et al., 2020; Al-Humaidi et al., 2021; Desai et al., 2021; Rommasi and Esfandiari, 2021; Giri et al., 2023). However, the cellular entry of nanocarriers is influenced by dynamic interactions between the carriers and the membrane. Therefore, carriers should be retrieved in the middle of the endocytic pathway, a phenomenon known as “endosomal escape” (Kim et al., 2019). Intracellular trafficking refers to the transport of cargo from the cell surface to its final destination within cellular compartments after cellular internalisation. NPs can be transported to various intracellular components. After cellular internalisation, NPs are confined within a membrane-lined vesicle, such as endosomes (Donahue et al., 2019). The partial cargo is transported to its destination by early endosome. Recycling endosomes with part of the cargo transports it to the plasma membrane. During maturation and differentiation, late endosomes come from early endosomes that integrate with lysosomes to form endolysosomal vesicles, and hydrolytic enzymes contained within these vesicles degrade the trapped NPs. Another intracellular degradation pathway that plays an important role in deciding the intracellular fate of NPs is autophagy. During autophagy, cytoplasmic contents are engulfed by autophagosomes and delivered to lysosomes to be degraded and recycled (Foroozandeh and Aziz, 2018). For accurate detection of endosomal escape, numerous nucleic acid- and peptide-based self-assembling nanomaterials, micelles, and peptide-conjugated QDs have been developed for intracellular delivery (Ruan et al., 2007; Kim et al., 2019; Yin et al., 2019).

Controlled drug release refers to the release of drugs during circulation. It contributes to the effective functional design and safe application of nanomaterials. Altogether, drug release rates, PK properties, and extravasation should be considered when designing NDDSs for achieving optimal outcomes. External stimulation may produce tailored drug-release profiles with temporal and spatial control (Meng et al., 2017; Yoon et al., 2020). Undifferentiated contact of NPs with physiological fluids results in the formation of protein corona that potentially shields surface functionalities (such as targeting ligands) and impedes drug release (Cai and Chen, 2019). In particular, targeted drug delivery plays an important role in stimulating responsive drug release (Chen et al., 2017). Given that the human body is a complex environment, further investigation is warranted to verify the effects of the *in vivo* release of drugs from NPs. Altogether, the therapeutic efficacy of NDDSs can be improved by selecting an optimal drug-to-carrier ratio and an adequate dosage of targeting ligands, thereby enhancing targeting ability and facilitating controlled drug release.

#### 4.2.7 Nanotechnology as an emerging field in BC research: progress and challenges

NDDSs can reduce systemic toxicity, realize efficient tumour targeting, have high bioavailability and drug solubility, and facilitate controlled drug release, thereby improving therapeutic outcomes (Din et al., 2017; Patra et al., 2018). To date, research in the field of nanomedicine has almost exclusively paid attention to tumour-targeted drug delivery. Nanocarriers that target tumours based on the EPR effect have been rapidly developed (Matsumura and Maeda, 1986). Many nanocarrier-based therapies have shown significant therapeutic efficacy in clinical settings (Khan et al., 2022; Park et al., 2022).

**FIGURE 14:**
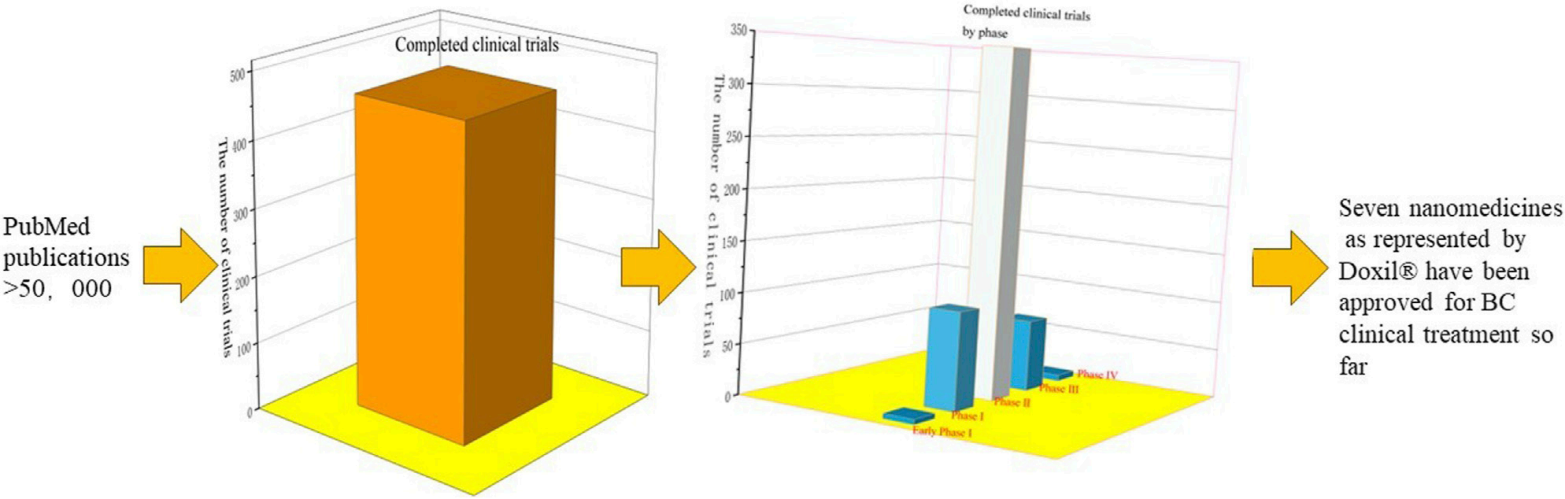
Data include papers published by PubMed and clinical trials registered at Clinicaltrials.gov. The number of publications indexed in PubMed >5,000; 465 studies were clinical trials (search was performed 1 July 2023) by querying Clinicaltrials.gov. The distribution of clinical trials with respect to the clinical trial phases demonstrated that the majority are in Phase Ⅱ (early Phase Ⅰ,5; Phase Ⅰ,97; Phase Ⅱ, 350; Phase Ⅲ, 72; Phase Ⅳ, 6, respectively). Seven nanomedicines have been approved for BC treatment.

As shown in Figure 14, the data included papers published by PubMed and clinical trials registered at Clinicaltrials.gov. They were identified using the following keywords: “Polymer OR Liposome OR Metal OR Quantum Dots OR Gold OR Superparamagnetic Iron Oxide OR Silver OR Carbon OR Mesoporous Silica OR Protein OR Dendrimer OR Micelle OR Nanoparticle” AND “Breast Neoplasm” OR “Breast Cancer.” The specific keywords for nano-formulations were chosen as they represented the most nanomedicines assessed in clinical trials. Specific keywords for nano-formulations were selected as they represented the large majority of NDDSs assessed in clinical trials. A total of 465 clinical trials were identified (as of 1 July 2023). The distribution of clinical trials with respect to the clinical trial phases demonstrated that the majority are in Phase Ⅱ. To date, seven nano-formulations represented by Doxil^®^ have been approved for the clinical treatment of BC (Jiang et al., 2022).

The aforementioned findings suggest an increasing interest in developing next-generation nanocarriers with unique properties such as active tumour targeting and TME responsiveness. The use of active targeting for improving DDSs efficiency has revolutionised cancer therapy. In active targeting, NDDSs selectively interact with the specific overexpressed antigens or receptors on the tumour cell membrane, enhancing their cellular uptake (Ahuja et al., 2021; Feng et al., 2021). NDDSs can exhibit a dramatic change in specific properties in response to characteristics of TME such as pH, temperature, light, reduction/oxidation, glutathione levels, and an abundance of certain enzymes (Uthaman et al., 2018). To date, various NPs also have been used in the treatment of BC, including liposome NPs (Nowroozi et al., 2018; Abumanhal-Masarweh et al., 2019; Le Rhun et al., 2020; Liu et al., 2022), SLNs (Wang et al., 2018), PNPs (Soltantabar et al., 2020), metal-based NPs (gold NPs) (Jafarizad et al., 2017), SPIONs (Poller et al., 2017), QDs (Sun et al., 2018)), carbon-based NPs (Garriga et al., 2020), MSNs (Zhao et al., 2018), protein-based NPs (Esfandiari et al., 2016), dendrimers NPs (Jain et al., 2019), and biological components such as exosomes (Al-Humaidi et al., 2021). With respect to SDDS, nano-scale hybrid liposomes coated with magnetoliposomes have been used for intracellular magnetic targeting in BC (Plassat et al., 2011).

Furthermore, nanotechnology may improve the therapeutic efficacy of radiotherapy and guide surgical tumour resection (Giri et al., 2023). PEGylated doxorubicin liposomal formulation (Doxil^®^/Caelyx^®^) was approved by the FDA in 1995. This formulation can be used to treat BC. Moreover, much research in this field has already led to the completion of dozens of clinical trials. Nanomedicine can improve the quality of life of patients by reducing adverse effects (Melchor-Martínez et al., 2021). For example, Abraxane™ (protein-bound PTX, approved by the FDA in 2005) has demonstrated significantly higher efficacy than standard PTX in improving the outcomes of patients with metastatic BC. In addition, the efficacy of passively targeted nanocarriers is limited owing to the heterogeneity of the EPR effect and the physiological barriers associated with it. Recently, Lee et al. (2017) used ^64^Cu-labelled HER2-targeted liposomes and PET/CT to assess the EPR effect with HER2+ metastatic BC in 19 patients.

Despite significant advances in nanotechnology, several challenges are associated with the development of NPs. The EPR effect and therapeutic outcomes of NDDSs are determined by multiple biological factors such as NP–protein interactions, blood circulation, extravasation into and interaction with TME, heterogeneity of EPR effect, TME-responsive delivery, and NP properties (e.g., size, geometry, and surface features). Furthermore, it is difficult to predict the safety and efficacy of NDDSs in humans based on preclinical animal models. *In vitro* and *in vivo* models of BC cannot accurately mimic the biological or clinical conditions of interest. On the other hand, selecting a target for actively targeted NCs is based on “classical” biomarkers (e.g., HER2 for BC). Actively targeted NCs can offer great advantages in treating BC. However, challenges presented by physiological barriers and MDR remain. Future studies should focus on developing optimal delivery systems for specific natural products (Yap et al., 2021). In addition, protein corona in the physiological environment, its consequences for biodistribution, and toxicity should be considered (Rosenblum et al., 2018). As shown in Figure 14, only a few nanomedicines reach clinical trials and market approval. More than 50,000 scientific research articles related to nanomedicine were published by the end of 2022; however, only seven nano-formulations have been approved for the clinical treatment of BC to date (Jiang et al., 2022). None of the active targeting nanocarriers have made advances in clinical trials (Rosenblum et al., 2018). The introduction of new DDSs is slow and expensive because clinical studies require time for rigorous assessment of safety and efficacy.

#### 4.2.8 Future perspectives and opportunities

In this section, we summarise the seven core research themes. The application of nanotechnology in drug discovery and delivery offers novel strategies for overcoming the challenges associated with the traditional treatment of BC. We used CiteSpace to determine the future directions of research in the field of NDDS-based BC treatment. Keyword and reference bursts revealed highly cited articles related to NP-based treatment of metastatic BC (Cheng et al., 2022a). Keywords (“green synthesis,” “optimization,” “proliferation,” and “molecular mechanisms”) have recently received substantial attention. The five important articles related to these keywords were Hossen et al. (2019) (strength, 3.07), Rizvi and Saleh (2018) (strength 4), Patra et al. (2018) (strength 4.34), Golombek et al. (2018) (strength 4.93), and Rosenblum et al. (2018) (strength 6.17) (Figures 7D, 9E).

“Green synthesis” results in the production of NPs that are non-toxic, eco-friendly, economical, reproducible, and easily amplified and have well-defined morphology (Khalid et al., 2017; Fadeel et al., 2021). Therefore, NPs based on natural products and microorganisms have gained considerable attention in the field of NDDS-based BC therapy (Nasir et al., 2021). Rahimi et al. (2019) used a green synthesis method to prepare a novel amphoteric calix [4] arene (Calix) macrocycle coated on the surface of Fe_3_O_4_ magnetic nanoparticles and used as a magnetic nanocarrier for simultaneous delivery of Dox and methotrexate in MCF7 cells. “Optimization,” frequently mentioned as “formulation optimization,” refers to the modification of an old nanocarrier design to improve its targeting and drug delivery efficiencies. Oseni et al. (2021) encapsulated andrographolide in PLGA nanoparticles via emulsion solvent evaporation, which improved therapeutic efficacy in a metastatic BC cell line. In addition, NDDSs can be optimised using components such as nanovesicles (Kassem et al., 2018). Alhakamy et al. (2022) used these to assess the cellular uptake of quercetin and its inhibitory effects on MCF-7 cells. “Proliferation” refers to anticancer activities through the inhibition of cell proliferation against BC in *in vitro* research, providing a novel approach nanodrug carrier system (Yewale et al., 2018; Sun et al., 2021). Many researchers have investigated the molecular mechanisms underlying the anti-tumour effects of nano-formulations and NDDSs, especially for developing new and smart NDDSs (Hossen et al., 2019; Cheng et al., 2021; Yap et al., 2021).

As mentioned in “Nanotechnology as an emerging field in BC therapy: progress and challenges,” the clinical translation of NDDSs is hampered. According to reference bursts, overcoming the challenges associated with drug delivery is a novel research direction (Patra et al., 2018; Rizvi and Saleh, 2018). Researchers have addressed these challenges by improving the design of NDDSs. SDDSs that can overcome multiple drug delivery barriers represent a promising tool for improving therapeutic efficacy. The development of SDDSs is a research hotspot in the field of materials science and pharmacy. Smart drug carriers will have a huge impact on the clinical treatment of cancer in the near future (Hossen et al., 2019).

According to reference bursts, “progress and challenges toward targeted delivery of cancer therapeutics” is another hot issue in the frontier (Rosenblum et al., 2018). Selecting a target for actively targeted NCs is based on “classical” or disease biomarkers (e.g., HER2 for BC). Actively targeted NCs can offer great advantages in treating BC. However, challenges presented by physiological barriers and MDR remain to be overcome. Future studies should focus on developing optimal delivery systems for specific natural products (Yap et al., 2021). In addition, consideration should be given to the formation of protein corona in the physiological environment and to the efficacy, biodistribution, and toxicity of drugs (Rosenblum et al., 2018).

One research direction is assessing the heterogeneity of the EPR effect, first described in 1986 (Matsumura and Maeda, 1986; Golombek et al., 2018). The clinical outcome of nanomedicine is not as good as anticipated on the basis of preclinical results, suggesting that many nano-formulations fail to produce the desired effects (such as tumour accumulation) and question the existence of the EPR effect (Nichols and Bae, 2014). This effect has been demonstrated to be valid and reproducible in all tested animal models; however, animal tumour models do not accurately mimic the TME of human patients. Moreover, the poor quality and reliability of published preclinical studies impede the clinical development of nano-formulations (Duncan, 2017) with a failure rate of 90%–95%. The failure of Phase II–III clinical trials can be attributed to the lack of efficacy (52%) and safety (24%) as well as strategic (15%), commercial (6%), and operational (3%) reasons (Harrison, 2016). Possible solutions may include the selection of an appropriate pathological variety that suits specific nano-formulations, followed by the establishment of an ideal animal model that best mimics the human cancer type. Selecting the right set of patients and treating them with the correct nano-formulations are key steps to successful clinical translation. Therefore, when aiming to develop nano-formulations for clinical use, the heterogeneity of the EPR effect should be considered and strategies should be developed to overcome this obstacle. Future studies should focus on developing a combination of therapies that potentiate the EPR effect and diagnostic protocols that enable visualization and quantification of the extent of the EPR effect in individual patients (Zhao et al., 2017). Perhaps imaging EPR-based tumour targeting may provide such evidence and quantification of the EPR effect in metastatic BC (Zhang et al., 2017).

Although this bibliometric study visualized the depth of the systematic review, it has several limitations. First, we provided an overview of the evolution of NDDSs in BC treatment. We comprehensively reviewed ongoing developments and determined future research directions. This meant that we were limited to data held in specific databases, which were inevitably biased. Second, data were downloaded from the WOSCC database owing to the format constraints of the bibliometric analysis software. Therefore, articles present in other databases were missed, and those with significant impact may not have been included. In addition, this study included publications only from 2012 to 2022. Third, we extracted original research and review articles published in English and did not include articles published in other languages and adhering to non-research types, which may have led to some omissions. In addition, a uniform algorithm is not available for optimising parameters in CiteSpace and VOSviewer for bibliometric analysis. The outputs may vary slightly, with different settings leading to confusion among readers. Fourth, an accurate description of the diagnostic and therapeutic strategies of BC may apply to a certain group of people but differ internationally. Finally, the past 20 years have seen rapid development in NDDSs. Nonetheless, big data provides valuable insights and guides decision-making in any domain. Researchers can gain an in-depth understanding of current bottlenecks and the developmental status of the field of NDDSs via big data.

## 5 Conclusion

To the best of our knowledge, this study is the first to conduct a bibliometric analysis to investigate research trends in the field of NDDS-based BC treatment. BC is a life-threatening disease among women. With the continuous development of nanotechnology, NDDSs have emerged as promising therapeutic agents for BC. They have demonstrated improved efficacy in clinical treatment while reducing systemic toxicity, realising efficient tumour targeting, increasing drug solubility, and facilitating controlled drug release (Din et al., 2017; Patra et al., 2018). To date, only a few nano-formulations have been successfully translated into clinical practice and approved for commercial use. An in-depth understanding of the challenges associated with the use of NDDS-based BC treatment is required to promote the clinical translation of nano-formulations. 1) Establishing *in vitro* and *in vivo* BC models should be improved to accurately mimic the pathological and clinical conditions of interest. Parameters such as the EPR effect, the immune status of the host, and the characteristics of TME should be considered. *In vitro* cell culture strategies (advanced 3D cell cultures, cell co-cultures, bioprinting, patient-derived cells, and microfluidic systems) may provide new hope for overcoming the existing limitation (Boix-Montesinos et al., 2021). 2) Tumour metastasis and MDR are dynamic processes and contain multiple targeting sites. To address this heterogeneity, combination or multi-target therapies and efficient NDDSs should be developed. 3) The application of NDDSs is limited by several factors. The following questions should be addressed for the successful clinical translation of NDDSs: overcoming the heterogeneity of the EPR effect and the physiological barriers in systemic NPs delivery; improving the manufacturing process for large-scale production of reproducible, high-quality nanomaterials. Despite increased interest and investment in the field, the introduction of new drug delivery formulations (active tumour-targeting strategies) and novel NDDSs remains slow-paced and expensive because their clinical translation and commercial approval require time to rigorously assess safety and efficacy.

In conclusion, NDDSs have good application value in the treatment of BC. Personalised treatment and risk assessment can be combined with NPs to promote the development of effective therapeutic strategies for BC. These efforts will accelerate clinical translation and benefit patients by improving survival. We speculate that nanomedicine will overcome these limitations. Therefore, the future of nanomedicine is promising.

## Data Availability

The raw data supporting the conclusions of this article will be made available by the authors, without undue reservation.
