# Supplementary material for: Current perspectives and trends in nanoparticle drug delivery systems in breast cancer: bibliometric analysis and review
Source: Front Bioeng Biotechnol. 2023 Sep 12;11:1253048. doi: 10.3389/fbioe.2023.1253048 (PMC10523396; doi:10.3389/fbioe.2023.1253048)
Supplement: Supplementary file 1 [file DataSheet1.docx]

supplementary material

Figure S1A showed the procedure for data retrieval and collection

Figure S1B Research framework

Figure S2 The landscape view was generated based on 1752 publications from 2012 to 2022


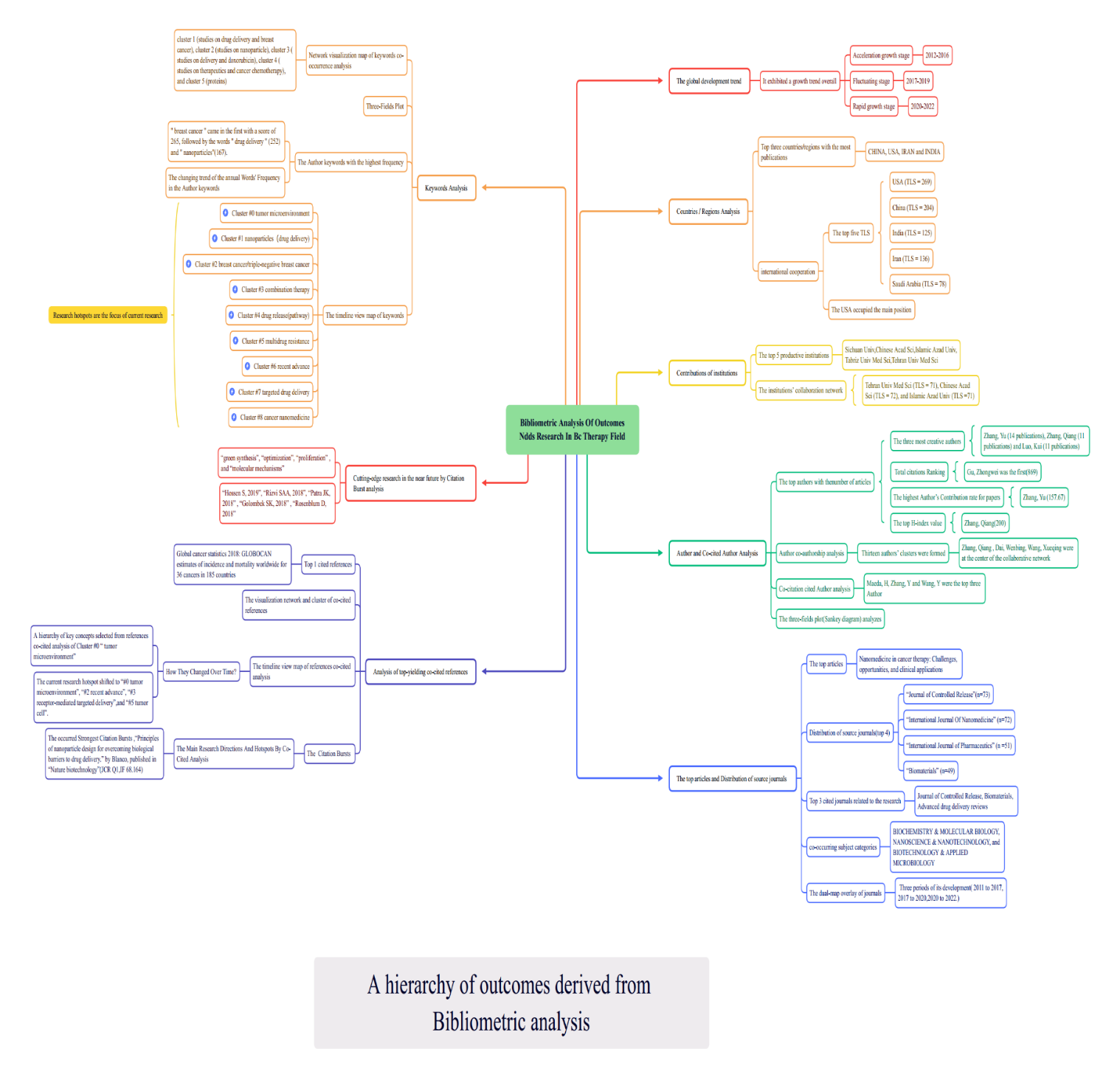


Figure S3 The timeline view of the landmark in adjuvant and neoadjuvant chemotherapy used in BC clinical trials (Waks and Winer, 2019)


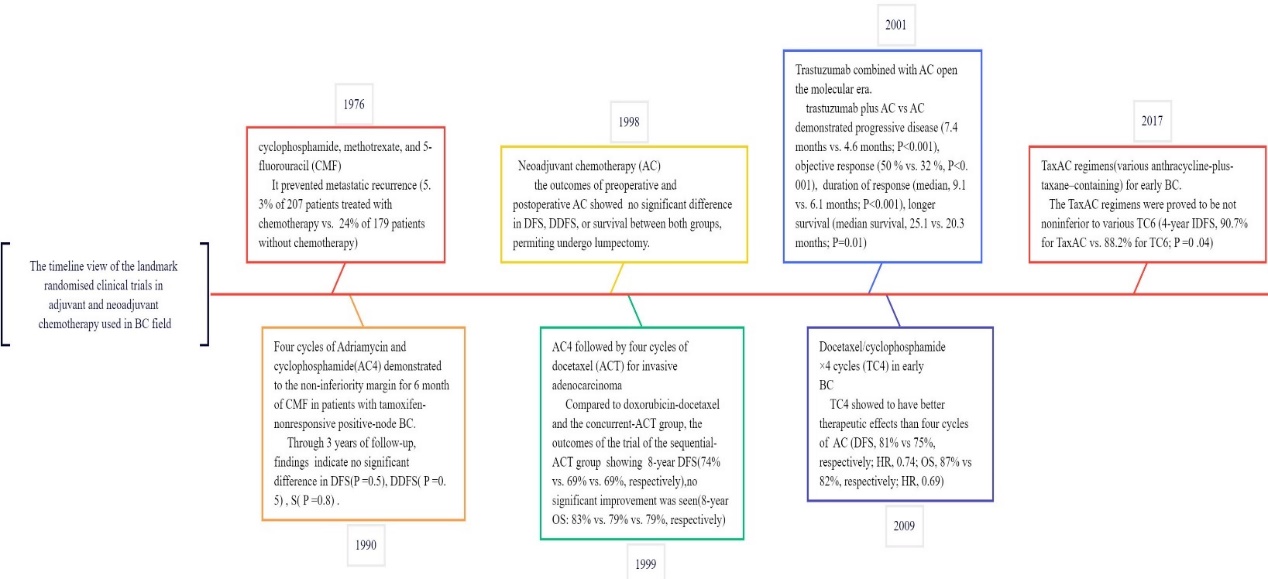


Table S1 Top 10 countries/regions with the most publications

| Ranking | Countries /Regions | Publications | SCP | MCP | Percentage | Total Citations | TLS |
| --- | --- | --- | --- | --- | --- | --- | --- |
| 1 | CHINA | 1835 | 517 | 86 | 36.69 | 20906 | 204 |
| 2 | USA | 870 | 148 | 71 | 17.39 | 13195 | 269 |
| 3 | IRAN | 857 | 141 | 53 | 17.13 | 3923 | 136 |
| 4 | INDIA | 503 | 139 | 40 | 10.06 | 4284 | 125 |
| 5 | SOUTH KOREA | 217 | 46 | 23 | 4.34 | 2000 | 78 |
| 6 | ITALY | 187 | 30 | 15 | 3.74 | 1561 | 66 |
| 7 | SAUDI ARABIA | 150 | 9 | 18 | 3.00 | 344 | 105 |
| 8 | TURKEY | 149 | 27 | 13 | 2.98 | 493 | 48 |
| 9 | EGYPT | 118 | 18 | 8 | 2.36 | 678 | 52 |
| 10 | CANADA | 116 | 18 | 9 | 2.32 | 1563 | 49 |

Table S2 The top 10 productive institutions ranked by the numbers of publications

| Rank | Institutions | H-index | Country | Total Citations | Avg. citations | TLS |
| --- | --- | --- | --- | --- | --- | --- |
| 1 | Sichuan Univ | 30 | CHINA | 2819 | 43.37 | 25 |
| 2 | Chinese Acad Sci | 37 | CHINA | 3464 | 72.17 | 72 |
| 3 | Islamic Azad Univ | 15 | IRAN | 663 | 15.42 | 71 |
| 4 | Tabriz Univ Med Sci | 22 | IRAN | 1192 | 27.72 | 41 |
| 5 | Tehran Univ Med Sci | 20 | IRAN | 978 | 23.85 | 71 |
| 6 | Shanghai Jiao Tong Univ | 19 | CHINA | 1096 | 29.62 | 41 |
| 7 | Shenyang Pharmaceut Univ | 17 | CHINA | 780 | 24.375 | 23 |
| 8 | China Pharmaceut Univ | 20 | CHINA | 1368 | 44.13 | 19 |
| 9 | Fudan Univ | 16 | CHINA | 1099 | 37.90 | 23 |
| 10 | King Abdulaziz Univ | 12 | Saudi Arabia | 814 | 33.92 | 32 |

Table S3 Top 10 authors with the highest number of articles in The Author Collaborated.

| Ranking | Author | Total citations | Author’s Contribution rate(100%) | Publication(WOSCC) | Total citations(WOSCC) | H-index(WOSCC) | Institutions |
| --- | --- | --- | --- | --- | --- | --- | --- |
| 1 | Zhang, Yu | 737 | 157.67 | 137 | 2,109 | 21 | Chinese Acad Sci |
| 2 | Zhang, Qiang | 821 | 59.39 | 2291 | 22,543 | 200 | Sch Pharmaceut Sci |
| 3 | Luo, Kui | 822 | 75.67 | [186](https://www.webofscience.com/wos/woscc/general-summary?queryJson=%5B%7B%22rowField%22:%22DXNG%22,%22rowText%22:%2211354665%22%7D%5D&displayJson=%7B%22key%22:%22author%22,%22icon%22:%22author%22,%22params%22:%7B%22name%22:%22Luo,%20Kui%22%7D%7D) | [4,020](https://www.webofscience.com/wos/woscc/general-summary?queryJson=%5B%7B%22rowField%22:%22DXNG%22,%22rowText%22:%2211354665%22%7D%5D&displayJson=%7B%22key%22:%22author%22,%22icon%22:%22author%22,%22params%22:%7B%22name%22:%22Luo,%20Kui%22%7D%7D) | 46 | Sichuan Univ |
| 4 | Gu, Zhongwei | 869 | 60.48 | 435 | 11,200 | 63 | Sichuan Univ |
| 5 | Ramezani, Mohammad | 261 | 91.62 | 420 | 9,201 | 63 | Mashhad Univ Med Sci |
| 6 | Alhakamy, Nabil A | 58 | 137.68 | 220 | 1,751 | 24 | King Abdulaziz Univ |
| 7 | Wang, Xueqing | 376 | 42.56 | 342 | 7,783 | 52 | Sch Pharmaceut Sci |
| 8 | Zhang, Hua | 355 | 77.05 | [127](https://www.webofscience.com/wos/woscc/general-summary?queryJson=%5B%7B%22rowField%22:%22DXNG%22,%22rowText%22:%2218428187%22%7D%5D&displayJson=%7B%22key%22:%22author%22,%22icon%22:%22author%22,%22params%22:%7B%22name%22:%22Zhang,%20Hua%22%7D%7D) | 3,483 | 36 | Sch Pharmaceut Sci |
| 9 | Li, Yaping | 448 | 46.44 | [662](https://www.webofscience.com/wos/woscc/general-summary?queryJson=%5B%7B%22rowField%22:%22DXNG%22,%22rowText%22:%2241561197%22%7D%5D&displayJson=%7B%22key%22:%22author%22,%22icon%22:%22author%22,%22params%22:%7B%22name%22:%22Li,%20Yaping%22%7D%7D) | 17,107 | 74 | Chinese Acad Sci |
| 10 | Sun, Jin | 135 | 50.95 | [2129](https://www.webofscience.com/wos/woscc/general-summary?queryJson=%5B%7B%22rowField%22:%22DXNG%22,%22rowText%22:%2232799870%22%7D%5D&displayJson=%7B%22key%22:%22author%22,%22icon%22:%22author%22,%22params%22:%7B%22name%22:%22Sun,%20Jin%22%7D%7D) | 31,291 | 90 | Shenyang Pharmaceut Univ |

Table S4A The top ten papers with the most citation on NDDS in BC（WOSCC）

| Title | First author | Journal | Published Year | Total  Citation | Funding Orgs(Y/N) | Main conclusion |
| --- | --- | --- | --- | --- | --- | --- |
| Nanomedicine in cancer therapy: challenges, opportunities, and clinical applications | Wicki, Andreas | *Journal of Controlled Release* | 2015 | 1260 | Y | Authors discuss the most advanced nanoparticles and targeted systems for frontier cancer treatment. They also emphasize the challenges faced and encourage clinical-translational science in nanoparticles research(Wicki et al., 2015). |
| Factors controlling the pharmacokinetics, biodistribution and intratumoral penetration of nanoparticles | Ernsting, Mark J.; | *Journal of Controlled Release* | 2013 | 667 | Y | The review shows us a picture of how the nanoparticles and biological factors influence the delivery of nanoparticles to tumors. The tumor microenvironment and imaging methods are also discussed so that patients can obtain the maximum benefit of nanomedicine (Ernsting et al., 2013). |
| Cancer Cell Membrane-Biomimetic Nanoparticles for Homologous-Targeting Dual-Modal Imaging and Photothermal Therapy | Chen, Ze | *ACS Applied Nano Materials* | 2016 | 525 | Y | The biomimetic nanoparticles (indocyanine green (ICG)-loaded and cancer cell membrane-coated nanoparticles, ICNPs) demonstrated specific homologous targeting to cancer cells with good monodispersity, preferable photothermal response and excellent fluorescence/photoacoustic (FL/PA) imaging properties. ICNPs significantly promoted cell endocytosis and homologous-targeting tumor accumulation in vivo(Chen et al., 2016). |
| EPR: evidence and fallacy | Nichols, Joseph W | *Journal of Controlled Release* | 2014 | 505 | Y | Clinical outcomes from nano-sized drug delivery systems have indicated that the enhanced permeability and retention is the controversy. And it could be attributable to high tumor interstitial fluid pressure, irregular vascular distribution, poor blood flow inside tumors, and the animal tumor models used(Nichols and Bae, 2014). |
| Codelivery of an optimal drug/siRNA combination using mesoporous silica nanoparticles to overcome drug resistance in breast cancer in vitro and in vivo | Meng, Huan | *ACS Applied Nano Materials* | 2013 | 458 | Y | The researchers reported a multifunctional mesoporous silica nanoparticle (MSNP) carrier to overcome doxorubicin(Dox) resistance in a multidrug resistant (MDR) human BC xenograft by co-delivering Dox and siRNA that targets the P-glycoprotein (Pgp) drug exporter(Meng et al., 2013). |
| Investigating the optimal size of anticancer nanomedicine | Tang, Li | *Proceedings of the National Academy of Sciences of the United States of America* | 2014 | 449 | Y | In this review, authors systematically evaluated the size-dependent biological profiles of three monodisperse drug-silica nanoconjugates (NCs; 20, 50, and 200 nm) through both experiments and mathematical modeling,which aimed to identify the optimal size for the most effective anticancer drug delivery(Tang et al., 2014). |
| Overcoming ABC transporter-mediated multidrug resistance: Molecular mechanisms and novel therapeutic drug strategies | Li, Wen | *Drug Resistance Updates* | 2016 | 433 | Y | In this review, authors examined the recent crystal structures of ABC proteins to depict the functionally important structural elements and the molecular mechanisms of various substrate interactions with the drug binding pocket, recent developments in anti-MDR strategies(Li et al., 2016). |
| Employment of enhanced permeability and retention effect (EPR): Nanoparticle-based precision tools for targeting of therapeutic and diagnostic agent in cancer | Kalyane, Dnyaneshwar | *Materials Science & Engineering C-Materials for Biological Applications* | 2019 | 405 | Y | The formation of leaky vessels and the poor lymphatic system is one of the characteristic features of cancer. Treating cancer by making use of the enhanced permeability and retention is increasing day-by-day. It generates multitudes of possibility to design novel anticancer therapeutics. Treating cancer by making use of the enhanced permeability and retention is increasing day-by-day and generates multitudes of possibility to design novel anticancer therapeutics(Kalyane et al., 2019). |
| Layer-by-Layer Nanoparticles for Systemic Codelivery of an Anticancer Drug and siRNA for Potential Triple-Negative Breast Cancer Treatment | Deng, Zhou J | *ACS Applied Nano Materials* | 2013 | 392 | Y | The researchers reported the strategy that provides a potential strategy to treat aggressive and resistant cancers. It was a modular platform for a broad range of controlled multidrug therapies customizable to the cancer type in a singular nanoparticle delivery system. The use of layer-by-layer films to modify a simple liposomal Dox delivery construct with a synergistic siRNA can lead to significant tumor reduction in the cancers (Deng et al., 2013). |
| A bioorthogonal system reveals antitumour immune function of pyroptosis | Wang, Qinyang | *NATURE* | 2020 | 382 | Y | The researchers reported a bioorthogonal chemical system, in which a cancer-imaging probed phenylalanine trifluoroborate (Phe-BF_3_) that can enter cells desilylates. It cleaves a designed linker that contains a silyl ether. This system enabled the controlled release of a drug from an antibody-drug conjugate in mice. When combined with nanoparticle-mediated delivery, desilylation catalysed by Phe-BF_3_ could release a client protein-including an active gasdermin-from a nanoparticle conjugate, selectively into tumour cells in mice(Wang et al., 2020). |

Table S4B The top 10 journals related to the research of NDDS in BC.

| Rank | Journal | Countries | Count | IF (2022) | | JCR (2022) | | H-index(2022) | | Percentage(%) |
| --- | --- | --- | --- | --- | --- | --- | --- | --- | --- | --- |
| 1. | Journal of Controlled Release | NETHERLANDS | 73 | | 11.467 | | Q1 | | 193 | 4.17% |
| 2. | International Journal Of Nanomedicine | NEW EALAND | 72 | | 7.033 | | Q1 | | 146 | 4.11% |
| 3. | International Journal of Pharmaceutics | NETHERLANDS | 51 | | 6.510 | | Q1 | | 217 | 2.91% |
| 4. | Biomaterials | NETHERLANDS | 49 | | 15.304 | | Q1 | | 248 | 2.80% |
| 5. | Colloids and Surfaces B-Biointerfaces | NETHERLANDS | 48 | | 5.999 | | Q1 | | 133 | 2.74% |
| 6. | ACS Applied Materials & Interfaces | UNITED STATES | 41 | | 10.383 | | Q1 | | 228 | 2.34% |
| 7. | Journal of Drug Delivery Science and Technology | FRANCE | 40 | | 5.062 | | Q2 | | 57 | 2.28% |
| 8. | Journal of Materials Chemistry B | ENGLAND | 40 | 7.571 | | Q1 | | 131 | | 2.28% |
| 9. | Materials Science & Engineering C-Materials for Biological Applications | NETHERLANDS | 36 | 8.457 | | Q1 | | 140 | | 2.05% |
| 10. | Pharmaceutics | Switzerland | 32 | 6.525 | | Q1 | | 83 | | 1.83% |

Table S4C Top ten cited journals related to the research

| Rank | Cited Journal | Countries | Citation | IF  (2022) | JCR  (2022) |
| --- | --- | --- | --- | --- | --- |
| 1 | JOURNAL OF CONTROLLED RELEASE | NETHERLANDS | 1355 | 11.467 | Q1 |
| 2 | BIOMATERIALS | NETHERLANDS | 1279 | 15.304 | Q1 |
| 3 | ADVANCED DRUG DELIVERY REVIEWS | NETHERLANDS | 1009 | 17.873 | Q1 |
| 4 | ACS NANO | UNITED STATES | 948 | 18.027 | Q1 |
| 5 | INT J NANOMED | NEW ZEALAND | 945 | 7.033 | Q1 |
| 6 | INTERNATIONAL JOURNAL OF PHARMACEUTICS | NETHERLANDS | 927 | 6.510 | Q1 |
| 7 | CANCER RESEARCH | UNITED STATES | 828 | 13.312 | Q1 |
| 8 | MOLECULAR PHARMACEUTICS | UNITED STATES | 810 | 5.364 | Q1 |
| 9 | PROCEEDINGS OF THE NATIONAL ACADEMY OF SCIENCES OF THE UNITED STATES OF AMERICA | UNITED STATES | 720 | 12.779 | Q1 |
| 10 | COLLOIDS AND SURFACES B-BIOINTERFACES | NETHERLANDS | 679 | 5.999 | Q1 |

Table S5A Top 10 cited references

| Title | First Author | Journal | JCR(2022) | IF (2022) | Counts | Year |
| --- | --- | --- | --- | --- | --- | --- |
| Global cancer statistics 2018: GLOBOCAN estimates of incidence and mortality worldwide for 36 cancers in 185 countries | Bray, F | CA: a cancer journal for clinicians | Q1 | 286.130 | 71 | 2018 |
| Cancer statistics | Siegel, R. | CA: a cancer journal for clinicians | Q1 | 286.130 | 67 | 2017 |
| Cancer nanomedicine: progress, challenges and opportunities | Shi, J | Nature reviews. Cancer | Q1 | 69.800 | 63 | 2017 |
| Analysis of nanoparticle delivery to tumours | Wilhelm, Stefan | Nature Reviews Materials | Q1 | 76.679 | 55 | 2016 |
| Nano based drug delivery systems: recent developments and future prospects. Journal of nanobiotechnology | Patra, J. K | Journal of nanobiotechnology | Q1 | 9.429 | 41 | 2018 |
| Global cancer statistics 2018: GLOBOCAN estimates of incidence and mortality worldwide for 36 cancers in 185 countries | Bray, F | CA: a cancer journal for clinicians | Q1 | 286.130 | 39 | 2018 |
| Principles of nanoparticle design for overcoming biological barriers to drug delivery | Blanco, E | Nature biotechnology | Q1 | 68.164 | 38 | 2015 |
| Nanocarrier for poorly water-soluble anticancer drugs--barriers of translation and solutions | Narvekar, M | AAPS PharmSciTech | Q3 | 4.026 | 29 | 2012 |
| Cancer nanotechnology: the impact of passive and active targeting in the era of modern cancer biology | Bertrand, N | Advanced drug delivery reviews | Q1 | 17.873 | 29 | 2014 |
| Stimuli-responsive nanocarriers for drug delivery | Mura, S | Nature materials | Q1 | 47.656 | 28 | 2013 |

Table S5B The Strongest Citation Bursts (Top 4)

| References | Title | Burst | Duration | Rang(2012– 2022) | Top 10 the highest cited references(Y/N) |
| --- | --- | --- | --- | --- | --- |
| Blanco E, 2015 | Principles of nanoparticle design for overcoming biological barriers to drug delivery | 12.87 | 2017-2020 | ▂▂▂▂▂▃▃▃▃▂▂ | Y |
| Barenholz Y, 2012 | Doxil® — The first FDA-approved nano-drug: Lessons learned | 12.39 | 2014-2017 | ▂▂▃▃▃▃▂▂▂▂▂ | N |
| Cabral H, 2011 | Accumulation of sub-100 nm polymeric micelles in poorly permeable tumours depends on size | 10.05 | 2014-2016 | ▂▂▃▃▃▂▂▂▂▂▂ | N |
| Wilhelm S, 2016 | Analysis of nanoparticle delivery to tumours | 9.88 | 2019-2020 | ▂▂▂▂▂▂▂▃▃▂▂ | Y |

Table S6 Summary of NPs and their properties used for BC treatment

| NPs Type | Solubility | Partic  le Size  (nm) | Clinical trials or translations | Advantages | Popular  drugs /gene |
| --- | --- | --- | --- | --- | --- |
| Polymer-based NPs | Highly  hydrophilic  and permeable | 100-300 | Phase III for Recurrent or Metastatic HER2-Negative BC | - Biodegradable - Biocompatible - Better thermal stability and mechanical properties. | PTX, Dox, Quercetin, trastuzumab and cisplatin |
| Liposomal NPs | Amphiphilic | 60-500 | - Phase III for leptomeningeal metastasis from BC; - PEGylated liposomal doxorubicin;   Approved | - The ease of preparation - Large-scale - Low-cost production - Biocompatibility - Biodegradability - Targetability - High stability - High drug loading capacity | DOX, PTX, siRNA |
| Metal-based NPs  (Gold NPs, superparamagnetic iron oxide NPs,quantum dots) | Hydrophilic | 10-1000 | - Phase Ⅰ for   Detection of cancerous sentinel lymph nodes in BC | - Unique catalytic, electrical, magnetic, optical - Simple surface - Thermal properties - Chemistry and functionalisation - Ease of synthesis | Dox, MTX, SMTX |
| Carbon-based NPs | Low solubility | 1-100 | N/A | - Structural diversity - Biocompatibility - Large surface area - Preferential tumour accumulation - Versatile surface functional groups | DOX PTX |
| Mesoporous Silica NPs | N/A | N/A | N/A | - Large surface area, pore volume - Simple fabrication - The capability to vary the pore size - High drug loading capacity - Biocompatibility Stability | DOX, siRNA |
| Protein-based NPs | N/A | N/A | - Phase I for advanced BC Nanoparticle albumin - Nanoparticle albumin - bound rapamycin (ABI-009) - bound paclitaxel (Abraxane®) | - Biocompatibility - Biodegradability - The highly ordered repetitive structures on the surface of viral NPs - Taking a short period of the synthesis of viral NPs | DOX Trastuzumab |
| Dendrimers | Solubility | N/A | - Phase II   PEGylated PLL dendrimer-based nanoformulation of SN- 38 (DEP® irinotecan)   - PEGylated PLL dendrimer-based nanoformulation of   docetaxel (DEP® docetaxel) | - Well-defined, homogeneous, and monodisperse structure - Bioavailability - High biological barrier penetrability - Stability | poly(ethylenimine)  (PEI) ,poly(amidoamine) (PAMAM) |

Table S7 List of therapeutic options for partial BC(Waks and Winer, 2019)

|  | Hormone Receptor (HR) +/ERBB2− | HER2-Positive BC | Triple-Negative BC |
| --- | --- | --- | --- |
| Typical systemic  therapies for  nonmetastatic disease  (agents, route,  and duration) | • Endocrine therapy (all patients):  • Tamoxifen, letrozole, anastrozole,  or exemestane  • Oral therapy  • 5-10 y  • Chemotherapy (some patients):  • Adriamycin/cyclophosphamide (AC)  • Adriamycin/cyclophosphamide/  paclitaxel (AC-T)  • Docetaxel/cyclophosphamide (TC)  • Intravenous therapy  • 12-20 wk | • Chemotherapy plus ERBB2-targeted  therapy (all patients):  • Paclitaxel/trastuzumab (TH)  • Adriamycin/cyclophosphamide/  paclitaxel/trastuzumab  ± pertuzumab (AC-TH±P)  • Docetaxel/carboplatin/trastuzumab  ± pertuzumab (TCH±P)  • Intravenous therapy  • 12-20 wk of chemotherapy;  1 y of ERBB2-targeted therapy  • Endocrine therapy (if also  hormone receptor positive)  • Tamoxifen, letrozole,  anastrozole, or exemestane  • Oral therapy  • 5-10 y | • Chemotherapy (all patients):  • A C  • AC-T  • T C  • Intravenous therapy  • 12-20 wk |
